# Social tectonics: Rapid organization of online COVID communities

**DOI:** 10.1371/journal.pone.0355369

**Published:** 2026-09-21

**Authors:** Katherine Van Koevering, Yiquan Hong, Jon Kleinberg

**Affiliations:** Department of Computer Science, Cornell University, Ithaca, New York, United States of America; Public Library of Science, UNITED KINGDOM OF GREAT BRITAIN AND NORTHERN IRELAND

## Abstract

The COVID-19 pandemic has been a large topic of interest on social media, with many different online communities addressing different dimensions of it. However, it differs from many other topics of comparable scale and complexity in that it appeared abruptly, over a short span of time for a topic of its size, and the online communities addressing it formed in a similarly short amount of time. We study the effects of this rapid self-organization using data on a nearly comprehensive set of COVID communities from Reddit, both as a substantive question about COVID discussions in themselves, and as a more general question about the form that the rapid self-assembly of several hundred online communities takes on a common overarching topic. Among our findings, we observe ways in which the newly-formed communities intersect with existing topical frames, including those around political, skeptic, and geographic organization. The effect of location was particularly strong, and geography formed the largest organizing theme in the communities, despite the global nature of the crisis. Overall, our work addresses questions in three aspects: what were people talking about, who were they talking about it with, and how did they self-organize these conversations?

## Introduction

Topics and communities exist in a form of symbiosis on the Internet, with large online communities forming around topics of shared interest, and online communities in turn shaping the structure of topical discussion. In general, the study of this process must necessarily start from a complex set of initial conditions, since broad topics almost always predate whichever Internet community has formed around them [1–3]. Viewed in this light, the COVID-19 pandemic has provided an unusual opportunity to perform a kind of analysis in which a large topic of interest appeared abruptly [4]. Not only was the disease itself a new topic, but there was of course very limited online discussion of even the more general issue that it represented — everyday life during a global pandemic. As a result, watching Internet activity concerned with COVID-19 lets us study the rapid self-organization of a collection of online communities around a large, multi-faceted topic of global interest.

We pursue this strategy through a large-scale study of COVID-19 communities on Reddit, beginning with a comprehensive enumeration and categorization of these communities, followed by an exploration of principles that emerge from their formation. One of the key points in this analysis is the way in which COVID-19, despite its newness as an issue, inevitably intersected a wide range of existing frames, including ideological, political, cultural, and geographic ones.

The role of geography shows up particularly strongly in the organization of COVID communities. For most people, COVID has been both a global phenomenon and a local one: many of the most pressing issues, including case counts, lockdown policies, vaccine availability, the closing and reopening of businesses and schools, and many other considerations were fundamentally local in nature; they varied considerably from one place to another. This locality manifested itself in social media as well, with online communities forming to focus on COVID in the context of specific cities, states, and countries. Such specialization is consistent with the ways in which online topics acquire strong geographic locality [5–7] more generally: despite the virtual nature of the medium, which makes communication possible in principle across large distances, the topics themselves are often of the most interest to people in a constrained geographic region.

In the case of COVID, this geographic specialization makes it possible to investigate questions about geographic variation in response to the pandemic. To begin with, we know through news reporting that different parts of the world, and different regions within large countries including the United States, had very different reactions to the pandemic, and appeared to develop different norms around the ways in which authoritative public health recommendations were handled. Can we see this reflected in differences among the discussions and the types of information shared in online COVID communities associated with different geographic locations?

Geographic specialization of communities also enables us to ask questions not just about the pandemic, but as a case study in how online media self-organizes at different geographic scales in response to rapidly-evolving global events. How was this geographic differentiation initiated online, and how early into the pandemic did it occur? How did it balance between “horizontal” specialization across similarly-sized regions and “vertical” specialization into a hierarchy of communities for cities, regions, countries, and the entire world? And did users tend to participate broadly across multiple geographically-focused communities over time, or did they remain focused on one or very few?

**The present work: COVID subreddits organization**

Here we address these questions using a large-scale dataset of COVID communities drawn from Reddit (reddit.com). Reddit has been one of the major loci of online COVID discussion, and its communities (*subreddits*, in the terminology of Reddit) exhibit the properties discussed above at a broad scale. Early in the pandemic, COVID subreddits formed both for a variety of topics closely related to COVID. We have assembled a dataset containing nearly all of these subreddits, collectively amounting to a significant proportion of the COVID discussion taking place on Reddit.

To address the questions discussed above, we organize our analysis of these COVID subreddits into two general themes of the dynamics of the communities and the structure of the communities. When one considers the dynamics of Reddit, there are two main components: the content and the users. We address users in terms of community membership and activity, investigating which communities users interacted with, how much, and when. For content, we focus on URLs, addressing how communities pulled in outside information and appealed to authority. For structure, we notice the striking impact of geography on the organization of the online ecosystem and investigate the role of geography in COVID discussion.

**Research question 1: How did COVID communities self-organize?**

- There is an intermediate organization of COVID as a topic on Reddit, in between the level of individual subreddits and the scale of all of COVID. We think of these as topical sub-categories, each consisting of a collection of subreddits that we call topic *classes*. Each of these functions as a kind of “tectonic unit” whose structure we can study, including the boundaries where they bump up against each other.
- Location plays a large role in COVID communities. More than half of the identified communities are explicitly linked to a location, and this accounts for nearly 40% of activity. 36% of users in our data set have participated in at least one geographically focused community.
- Organization of communities cemented quickly. Most communities were created within a few weeks of the declaration of the pandemic. The exceptions mostly related to vaccines and long COVID – events that happened later in the pandemic and provoked a response. We see little evidence of reorganization after this initial burst of activity. To continue the tectonic metaphor, we see rapid cooling and solidification of the basic units within a short time period, followed by much slower rates of change after that.

**Research Question 2: How do users divide themselves among these communities? What are the patterns underlying these affiliations?**

- One common theme among users is that users who participate in more subreddits, rather than spreading their activity more thinly among this large collection of subreddits, are much more active on a per-subreddit basis. In particular, for a user that participates in *n* subreddits, the amount of activity in their $k^{th}$ favorite subreddit will on average be higher than the amount of activity in the $(k-1{)}^{st}$ favorite subreddit for a user with n−1 subreddits. However, the average amount of activity in a user’s favorite community stands at approximately 40% of their total activity regardless of how many communities a user is part of. In fact, this percentage is stable even for 2nd and 3rd favorite subreddits at about 20% and 11%.
- Insularity varies widely across both communities and classes. The percentage of users that engage in other communities or classes can be as low as 15% and up to 80% or higher. Even communities within the same class can have wildly disparate insularity scores, although classes do seem to have tendencies overall. This suggests that there is no single ratio of modularity that would be effective for community detection in this case, presenting a methodological issue in terms of community detection and questioning long-standing practice of assuming modularity ratios are the same across communities within a data set.

**Research question 3: How does posted content vary across communities?**

- We emphasize that URLs are a very effective tool for understanding outside influence on a community. They are an efficient short-hand for how communities gather outside information and use it internally. They are also easily comparable between communities, as we see the same domains and URLs in use across our data set.
- By analyzing these URLs we can also quantify appeals to authority. Anti-Vax communities, for instance, reference the CDC and other US government websites far more than other communities, even those focused on discussing COVID as a disease.
- We also see a temporal shift in outside information. While social media, regional news, and even science domains see roughly steady use throughout the pandemic, more national and international news sites see a precipitous decline driven by a single class of community – geographically linked communities.

**Research question 4: How did the subset of geographically focused communities self-organize?**

- Using vote shares for the two main US political parties in the 2020 US Presidential election, US states with Democratic majorities tend to share more common domains, such as medxriv.org, than Republican states, which share more uncommon domains, such as thetennessean.com. Additionally, many of the domains that are most over represented in states with Democratic majorities correspond to scientific authorities, an interesting and fundamental social-media reflection of a partisan phenomenon that has appeared in survey data [8].
- Another interesting consequences of the rapid formation of the communities is the presence of *isomorphic* communities — different subreddits devoted to the same local geographic region. In cases where multiple subreddits are created for the same location, there is nearly always one dominant subreddit, and that is usually the first subreddit created.
- Additionally, users are more likely to join location based communities that are geographically close to each other, and communities that are close vertically – for instance to join a city that resides in a state they already discuss.

### Data

For our analysis, we use 434 COVID subreddits with data covering almost 2 years for each, from the beginning of 2020 to the end of 2022, which covers the vast majority of the active period for all subreddits. We pulled data from the pushshift dataset [9], which at the time of data collection was openly available and compliant with Reddit terms of service but has since shut down [At the time of analysis, in the beginning of 2023, this data was publicly available and was downloaded from pushshift.com during that period. Since then, Reddit has changed their terms of service and removed comprehensive public data dumps. Equivalent data is available at the Internet Archive, https://archive.org/download/pushshift_reddit_200506_to_202212. Due to these third-party restrictions,the authors do not intend to publish the dataset used in this study in any modified format,and refer to the Internet Archive.]. These were found by identifying all subreddits with the words ‘coronavirus’ or ‘covid’ in the name, only excluding subreddits with fewer than 200 subscribed users as of the end of 2022 and those that were clearly not related to the pandemic. We supplemented this with any communities we could find in other work on Covid on Reddit [10,11], such as China_Flu. Based on additional followup investigations using the Reddit search function and the “similar communities” function, this represents the vast majority of large, novel, COVID-explicit subreddits. Specifically, we used Reddit’s community search for the terms coronavirus, covid, and pandemic, and compared out list to the first 100 results. We found only a few missed communities, suggesting we had discovered most large relevant communities. We also went to the homepage of the most common subreddits in each category and checked the ‘related communities’ tab provided by Reddit and included any additional communities that fit our criteria, of which there were only a few, again suggesting this is a good sample of large Covid communities. Reddit was chosen for this analysis partially because of the unique, explicit labeling of COVID discussion communities by topic – each community is given a name and short description that describe the theme of the community. This gives ideal conditions to study community-level rather than just individual-level interactions. Reddit is also a common target of academic discourse, easily obtainable, and with known and well-documented biases (for instance largely American, male, and white) [12].

Subreddits were manually classified based on their name and description. Classes were organically derived from these descriptions by a collaboration of two researchers – one creating designations and another verifying classes. These classes gave a relatively low Fleiss’ Kappa score of 0.48. Subsequently, two experts researchers accessed the post/comment history of subreddits of high disagreement and came to a joint conclusion. Most disagreement was due to two effects: ambiguous sarcasm/joke subreddits and subreddits that spanned multiple categories, such as a subreddit that dealt with recovery from a COVID infection and emotional support during that recovery. The messy nature of these communities suggests difficulty in categorization, but small changes in the occupants of the categories do not appear to meaningfully effect most results. A few subreddits have since been deleted or marked private and they are given the Other designation. Location subreddits were additionally manually tagged with the location the represented, and given a **level** of either ‘country’, ‘state’, ‘city’, ‘state_f’ or ‘city_f’ to designate the vertical level of the location. These represented general country-specific subreddits, US state subreddits, US city subreddits, non-US state subreddits, and non-US city subreddits, respectively. In the case that a subreddit was ambiguous (e.x. Southern California), the level closest to the size of the region was specified. In this case, Southern California is considered state level. A European subreddit would be considered country level, since it is closer to a country than a top-level general or global subreddit.

### Overview of related work

This work is related to several branches of literature. **Reddit and Covid** In work that takes a more narrow view, Zhang et al [10] study the organization and evolution of a few of the largest COVID subreddits Ashford et al and Dow et al [11,13] consider misinformation and conspiracies about COVID online. Additional work looked at the long-term changes that the pandemic had on Reddit and the users of Reddit [4], concluding that change in both language and behavior were pervasive throughout the platform, and that the event ushered in a wave of new users that have continued to behave differently. This also supports treating covid subreddits as somewhat different in their behavior than typical topical-based subreddits and this broader structural transformation of Reddit is important context for interpreting the emergence and evolution of COVID-specific communities. This contet further muddies the waters of what novel behavior is specific to Covid and what novel behavior is more relevant to crises generally. In work that is more similar to ours in its geographic focus, Yan et al use Reddit to study the sentiment about vaccines in particular across cities in Canada [14]; we broaden the questions to a larger enumeration of topics and locations, and at multiple spatial scales. Aubin Le Quéré et al focus on the local news coverage of COVID on online communities, but do not analyze the communities [15] themselves. Dong et al. [16] complement this by examining community wellbeing and resilience in location-based subreddits during the pandemic, finding that expressions of solidarity and shared experience correlated with higher subjective wellbeing — although this focuses on communities that are not explicitly related to the pandemic.

**Covid Online.** The COVID-19 pandemic has been a global subject of intense focus on social media since its early stages [10,17], and it has exemplified many of the fundamental properties of social media usage during major world events. People have used social media for social support [18] and online sense-making, both of which are common practices during unfolding crises [19,20]. Online discussion about COVID has had strong political dimensions [21], and social media has been a source of COVID misinformation and conspiracy theories [11,13]. Recent work has also examined how users construct legitimacy and authority in COVID discourse. Beers et al. [22] show how anti-mask and anti-vaccine communities selectively cite official sources to construct “dueling consensuses.” – a concept we mirror in the use of URLs in skeptics communities. Harris et al. [23] find that antivaccine Twitter communities amplify the voices of “perceived experts,” while Efstratiou et al. [24] demonstrate how false consensus is manufactured through repeated citation and alignment. These dynamics are relevant to our findings on how Reddit users — including skeptics — appeal to institutional authority, often through strategic citation of government sources, but also in our results of their links to unusual and non-traditional media.

**Crises Online.** Generalizing beyond COVID, our work is also related to the study of social media use during crises [19,20]. In particular, the type of self-organization and collective sense-making through online interaction has been described in earlier work by Starbird et al as a kind of “verbal milling behavior” [25] to seek out and share information in the midst of uncertainty. Additional focus on social media as a tool for organizing information flow and responses in crises tend to focus on acute events, rather than a drawn out crisis like a pandemic [26,27]. These studies show social media as an essential tool for both the general public and responders, allowing for broader participation in information sharing, more local and specific information sharing, and increased danger of misinformation. See Park et al. [27] and Reuter et al. [28] for a more thorough review of social media during brief crises – a topic that has been popular for decades and is outside the scope of this short review.

**Online Geography.** Our work is also related to general efforts to map the spatial organization of different forms of online information. Berregan et al. note that Reddit is divided neatly into many geographical subreddits, which do seem to draw from local tendencies in their lexical footprint [29], while Bozarth et al. and Riley et al. look at the importance of these subreddits in relation to the spread of news URLs [30,31]. Other work has also looked at the geographic loci of search engine queries [5–7], and recent papers have studied the effectiveness of online activity at identifying hot-spots of COVID [32–35]. Our work builds on this by comparing how geographic references and spatial scales are invoked across both general and COVID-specific subreddits. The rapid collision of the digital and physical world has also spawned work on digital placemaking, attempting to understand how individuals derive a sense of place from the interaction of these worlds. This idea of digital placemaking complements the remarkable geographic tendencies seen in crisis response and social media in general. See Suzuki and Dillon for an overview of digital placemaking [36].

**Community Interaction.** Kumar et al. discuss conflicts between communities are hallmarked by the formation of echo-chambers, suggesting insularity as a negative trait [37], but this sort of conflict is pervasive across Reddit and can also be linked to users who seek separate communities to be social and anti-social in [38]. More recent work has expanded this view: Efstratiou et al. [39] show that echo chambers often coexist with hostile inter-group interactions, complicating the notion of ideological silos. Rollo et al. [40] map attention flow across political subreddits, identifying “bridge” users who connect otherwise siloed groups. This sense of conflict is highlighted in Covid communities with explicitly opposing view points, but also in how isolated communities in some categories are compared to others.

Hickey et al. [41] track user migration among hate subreddits, offering insight into how moderation, identity, and shifting interests shape user trajectories. Waller & Anderson [42] provide a broader framework for understanding how platform design influences social organization and polarization — relevant for interpreting Reddit’s affordances in shaping community interaction.

## COVID on Reddit

We classify the communities we study into 19 classes,described in Table 1. As with all Reddit communities, these classes describe the topics the communities are built around. It is worth noting that one category is larger than all of the rest put together – Location. With almost 300 communities dedicated to location specific COVID topics, this suggests that Reddit COVID discussion is strongly influenced by geography. Whatsmore, this category is so unusual that we find it necessary to do additional analysis separately. Most other categories have between one and twenty communities. The classification is based on the description the community has for itself as well as the name of the community.

**Table 1:** Subreddit class descriptions.

Activities During Covid	Discussing a normal or prior activity/task now done with COVID restrictions or impacts	21
Anti-Anti	Discussing and mocking skeptics	7
Anti-Lockdown	Discussing lockdown opposition or skepticism	3
Anti-Vax	Discussing vaccine opposition or skepticism	8
Disease Discussion	Discussing COVID virus, symptoms, or status or first-order effects of the disease	18
Economy	Discussing impacts on global economy and economic recovery	11
Emotional Support	Emotional support specific to COVID such as grief support	7
General	General discussion of COVID disease + effects without more specific purpose	11
Location	Location-specific discussion	282
Location+	Location-specific discussion w/restrictions	3
Location-Anti	Location-specific discussion for skeptics	1
Long Covid	Discussing long COVID	4
Memes	Memes for/about COVID	13
NSFW	not safe for work content specific to COVID	3
Official	r/Coronavirus: the official community	1
Other	communities w/o identifiable description or purpose	7
Recovery	Discussing experiencing or recovering from a COVID infection	15
Skeptics	Discussing skepticism of COVID or offical narratives	14
Vaccine Discussion	Discussing COVID vaccinations	5

Classes of COVID communities, along with description and subreddit count. These classes represent broad categories of topics for communities, where topics were determined by subreddit description – a short description written by the communities’ moderators. These classes were generated organically by one researcher, and then verified by a second. Note that r/Coronavirus was designated the official COVID community by Reddit and both had additional support from Reddit and was under additional scrutiny. Note that some classes may subsume others. For instance, Recovery may be considered a sub-class of Disease Discussion – in this case the most specific class was chosen.

### Communities

**Research question 1: How did COVID communities self-organize?**

**Fig 1:**
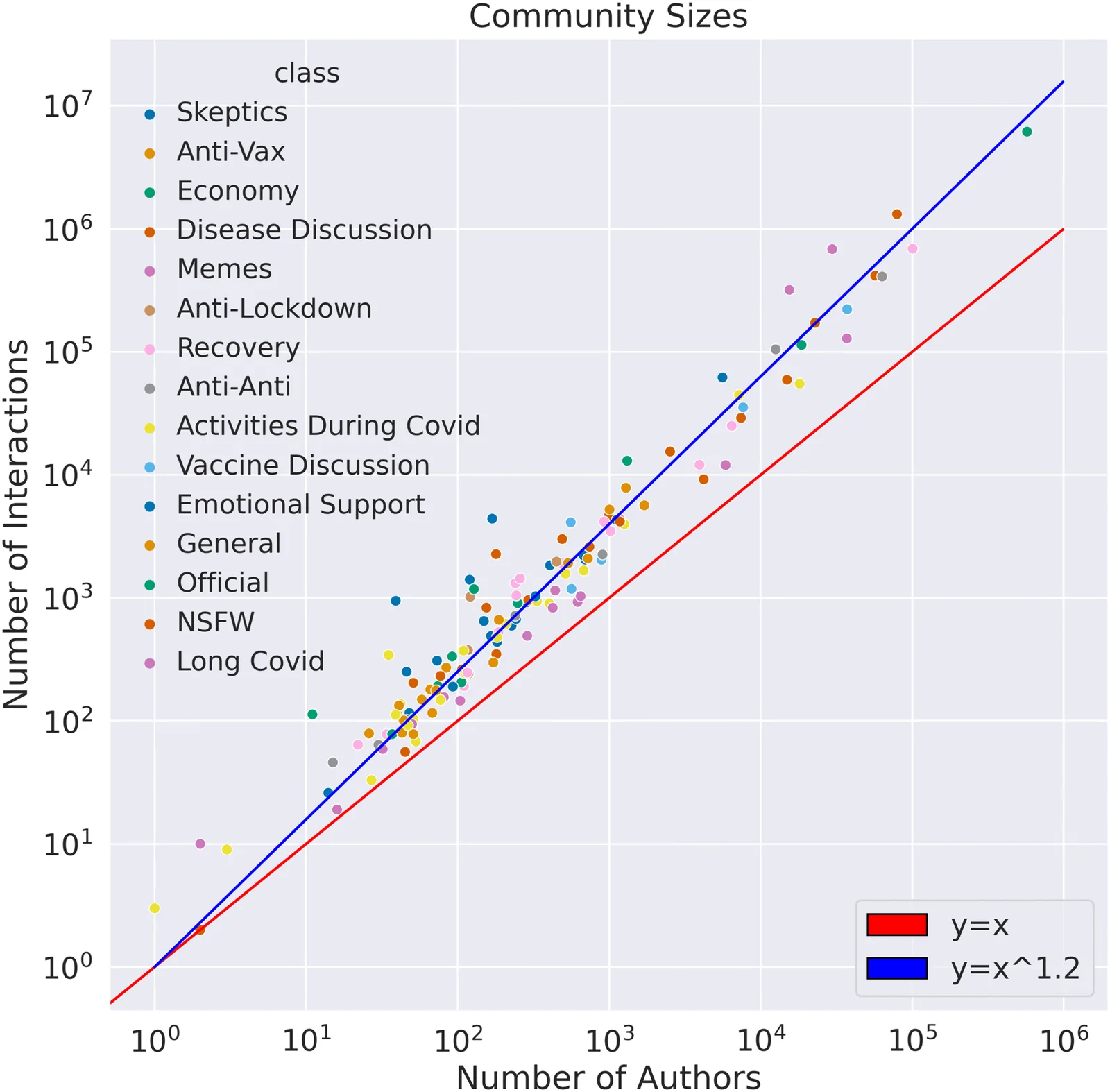
Sizes of non-location communities. Relative sizes of non-Location communities by both number of authors (user) and the number of overall posts/comments (interactions) on a log-log scale. Generally, these two measures are highly correlated [3,43]. The largest by far is r/Coronavirus – the Reddit designated official community. Note that larger communities tend to have more interactions per author. The red line is the diagonal, but the blue line is *y* = *x*^1.2^, suggesting that if two communities *A*,*B* have authors such that *A* has twice as many as *B*, we would expect *A* to have about 2.3 times as many interactions. This scale is important to remember when we discuss large and small subreddits, as size in terms of authorship and activity are not linearly, but logarithmically, related.

**Fig 2:**
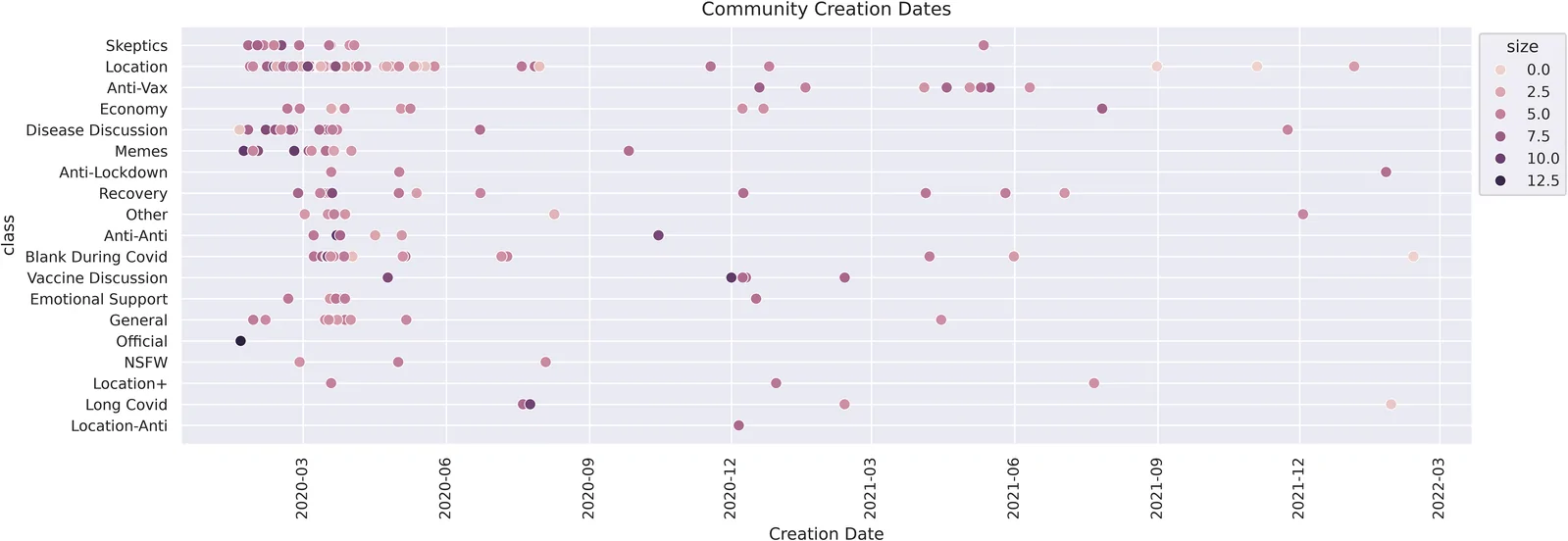
Dates of first post for subreddits. Dates of first post for communities by category, colored by size. Darker communities are larger by the end of our study period. Note that r/Coronavirus is a previously-established subreddit but had minimal activity prior to the pandemic. Note also the relative burstiness of different categories [44]. Most communities were created within the first few months of 2020, with only a few additional categories created later on, which agrees with prior research and reaffirms the possibility of temporal analysis as the history of most subreddits is quite similar [4]. Size is on a log scale in terms of number of interactions.

Reddit communities are user created, user moderated, and user organized. Thus, the creation and organization of these communities is a crowd-sourcing exercise, that describes how the larger Reddit community chose to organize the discussions about COVID. To understand these classes, we look both at their size Fig 1 and their creation dates Fig 2. We can see that there are communities of all sizes across all categories. Additionally, although size in terms of number of members and size in terms of number of posts/comments are very similar, larger communities tend to have sightly more interactions than authors, suggesting more active membership. Additionally, most communities have between 100 and 10000 authors/interactions. Creation dates also give us a snapshot of concerns throughout the pandemic. The first classes to have communities include Disease Discussion, Memes, Skeptics, and Location (note that r/Coronavirus existed prior to the pandemic, but was mostly dormant). A little later we see Economy, Recovery, Anti-Anti, Emotional Support, and Activities During Covid. Vaccine Discussion starts to become active in April 2020, but Anti-Vax communities don’t come online until 2021 (when additional Vaccine Discussion communities are created). Long Covid specific communities are created in July 2020. As for most classes, there seems to be a burst of communities created at soon as the topic becomes a concern, but additional communities continue to be created until nearly the end of our time period. This could suggest fragementation of the topic into sub-topics or fragmentation of the audience into sub-audiences, or a surge in popularity of the topic that requires additional space to discuss. This is further supported by the fact that the most active community in a class is rarely the first one created – suggesting that they are not merely overlapping topics but each community serves a different purpose.

**Fig 3:**
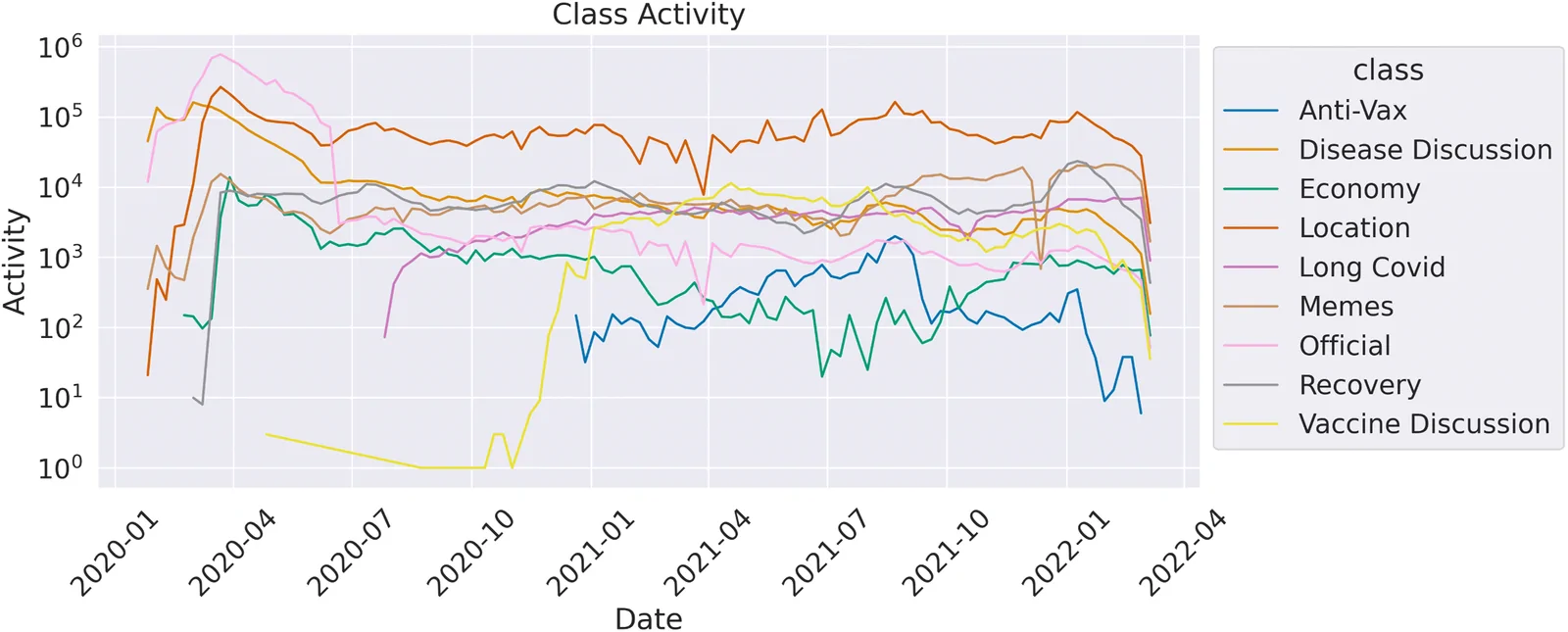
Weekly activity by category. Amount of weekly activity for various categories on a log scale against a timeline. Note that the most active classes are Official and Location for most of the time period. We also see spikes of activity at points that reflect a common understanding of the pandemic – Recovery lags behind a couple of months and Anti-Vax spikes shortly after Vaccine Discussion. This lends credence to our categories and data collection methods, as it affirms expectations on temporal popularity of various categories. The relative stability of topics, however, is also interesting with the context of most subreddits being created early on. The landscape of COVID on Reddit was established quickly and had few major changes – with the changes it did have mostly relating to changes external to Reddit such as vaccine development.

If we look at the activity for some of these classes in Fig 3, we see there is some overlap with this. Activity in Vaccine Discussion spikes at the end of 2020 and beginning of 2021, coinciding with additional communities in both Vaccine Discussion and Anti-Vax. Additionally, most classes experience an initial burst of activity and then a slow decline, reflecting the community creation burstiness and the rapid organization of classes and communities. Additionally, we see a precipitous drop in the activity of Official early on that roughly coincides with this initial burst of creation of communities, suggesting the initial fragmentation of r/Coronavirus into what became our classes – a fragmentation by topic as the need for additional space to discuss topics quickly overwhelmed the main existing community.

Given that each class may contain many communities, it is natural to wonder what the relationship between these communities is. In some cases, a single community stands out as far and away the largest in a class. In other cases, communities share users more equally (Table 2). For instance, the Long Covid class is dominated by a single community – r/covidlonghaulers, but the Location class has many communities of many sizes. There are a number of things that could influence this tendency. The Location class, by nature, has many locations that must necessarily be represented by different communities. It would make little sense to combine them. The Long Covid class does not have this property in the same way. However, while the Skeptics class is dominated by r/CoronavirusFOS, the Anti-Vax class, which might seem to have similar properties, does not have one stand-out community. This fracturing of a class could because of a split in the topics discussed (as in the case of Location) or a split in the users or both. While it is difficult to trace cause and effect for community creation, in the case of Anti-Vax it may be because of interference from Reddit for misinformation, as communities fractured based on community rules and speech guidelines.

**Table 2:** Dominant subreddits.

Class	Largest Community	% of Activity	Community Ratio
Long Covid	covidlonghaulers	1.00	309.47
Other	COVID19_commentary	0.98	138.26
Recovery	COVID19positive	0.93	27.57
Skeptics	CoronavirusFOS	0.81	14.41
Economy	CoronavirusRecession	0.88	8.74
Vaccine Discussion	CovidVaccinated	0.84	6.29
Memes	CoronavirusCirclejerk	0.82	5.33
Anti-Anti	CovIdiots	0.79	3.92
NSFW	CoronavirusNSFW	0.74	3.59
Disease Discussion	China_Flu	0.65	3.16
General	covidsupport	0.50	3.16
Emotional Support	InMemoriamCOVID	0.51	2.39
Anti-Lockdown	ImDoneWithCovid	0.59	1.93
Location	CoronavirusDownunder	0.28	1.77
Anti-Vax	CovidVaccineInjury	0.37	1.39
Activities During Covid	covidcookery	0.49	1.23
Location+	CoronavirusUKCasual	0.45	1.23

The percentage of activity of the largest community for each class except Location, and the ratio of the largest and second largest communities. Note that most classes are dominated by a single community and many are nearly entirely composed of 2–3 communities regardless of our search uncovering many more. This suggests that, for most categories, the needs of the users are met by a small number of communities despite many existing. This brings to mind a number of dynamics, including a struggle for relevancy among highly related topical communities as described by [45] or a hub-and-spoke system of large communities receiving most activity and a number of smaller, specialized communities filling more niche roles.

### Users

**Research Question 2: How do users divide themselves among these communities? What are the patterns underlying these affiliations?**

Many, although not most, of the users in our data set participate in multiple communities (Table 3). For the purposes of this work, we only are able to see user interactions with a subreddit – either through posting or commenting – but not viewing or subscribing. Thus, we only consider which communities users interact with directly. While we have attempted to remove the most proliferate bots, it is unlikely that we managed to eliminate all bot accounts. However, with a weighted random sample of user accounts, we find very few explicit bot accounts left in the data.

**Table 3:** Users in multiple classes/subreddits.

Count	Subreddits	Classes	Random Classes
1	958045	983341	1048662
2	176920	176020	145344
3	52854	48283	25372
4	20201	14180	5991
5	9009	4637	1416
6	4696	1547	324
7	2576	561	41
8	1440	145	2
9	868	66	1
10	548	31	0

Number of users who have participated in *n* subreddits or classes. As is standard in social media research [9], users interact in an exponential fashion, with more than half of users participating in exactly one community. Note that, for low values, the number of users in *x* classes closely tracks the number of users in *x* subreddits. This implies that, for users in few subreddits, the subreddits are generally all in different classes rather than the same class. That is, when joining multiple communities, most users join communities that are more different as opposed to communities that are very similar. However, as *x* increases this becomes less true – users begin to repeat classes. This suggests two patterns of user behavior – users who are more concentrated in community are less concentrated in class, whereas users who are less concentrated in community are more concentrated in class.This rule is to be expected at random, as users run out of classes to join, given our short and broad classification system, but is more pronounced in our data than expected at random. The final column shows the class distribution for a simulation where users choose *x* subreddits uniformly at random. Although the values are similar to the random simulation, the real world shows more users with high numbers of classes than would be expected at random, suggesting users are intentionally distributing themselves among multiple classes and part of the motivation for using a new subreddit may be wanting access to a new class.

**Fig 4:**
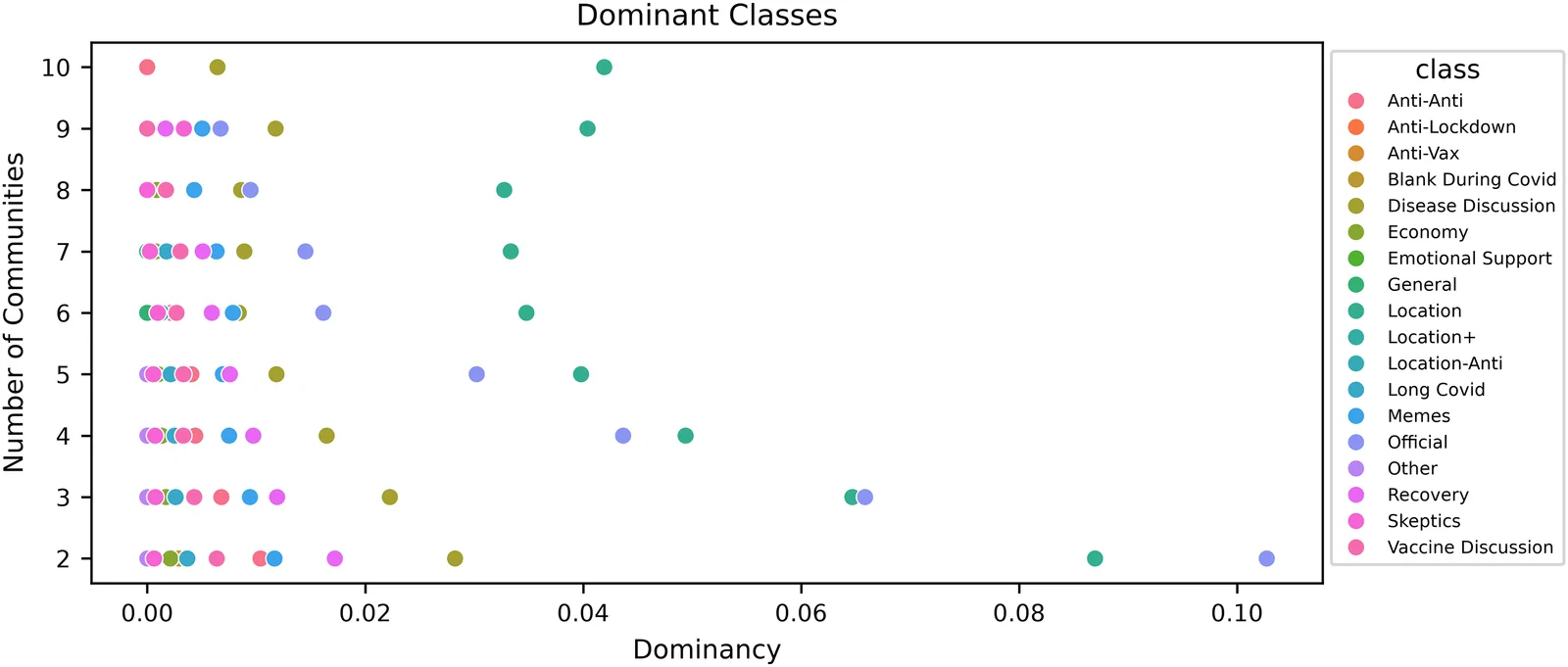
Users with dominant classes. Percentage of users with *y* active communities whose dominant class is a given type. For users with three or more active communities, the vast majority have Location as a dominant class. That is, users who are in at least 3 communities (a small percentage of users), spend almost all of their time in a geographically-linked subreddit and only some time in other communities – in particular they spend less time in r/Coronavirus than we would expect. For users with one or two active communities (the vast majority of users), the Official class is dominant (they spend most of their time in r/Coronavirus). This affirms the result that users with very few active subreddits are disproportionately active in r/Coronavirus – and thus more ‘casual’ users, the majority of users on Reddit, are more impacted by any changes in this subreddit, which is particularly noteworthy given the extra attention Reddit paid to this subreddit and its policies. This also may suggest the strategy of designating an official community was effective in reaching most users, but it could also be seen has perhaps having a limiting effect on growth of other communities as it concentrated casual users in one place.

We also can have a similar definition of dominancy for users. As a short-hand rule, let us say a user has a dominant community if their most active community is at least twice as active as their next most active community. That is, where they post at least twice as much in their favorite subreddit as their next favorite. By this definition, about 53% of users have a dominant community. By far the most common dominant community is r/Coronavirus, followed by r/China_Flu and r/COVID19positive. 55% of users have a dominant class by the same rule. As we can see in Fig 4, this dominant class is almost always either Official or Location. While the number of communities increases, the percentage of users with a dominant class decreases overall. However, the percentage of users with a dominant class of Location is not strictly decreasing, again cementing Location as an unusual class. This analysis of dominancy closely relates to prior work on community loyalty where aspects of the community (and the user) can inspire greater loyalty and thus more dominant communities [46].

**Fig 5:**
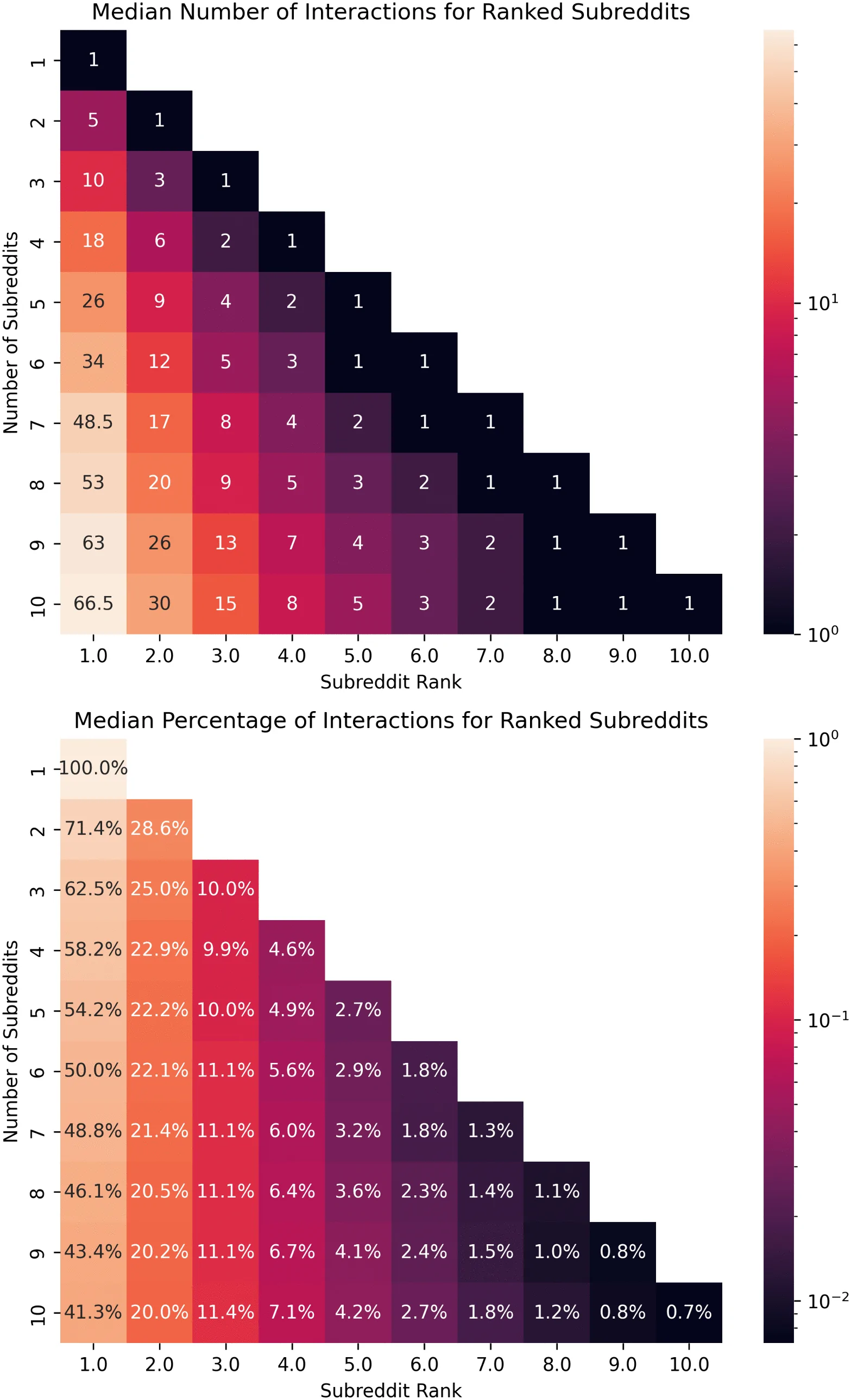
Median number of interactions (top) and percentage of interactions (bottom) with the $x^{th}$ most interacted-with subreddit for users that interact with *y* subreddits. The combination of users with more subreddits being more active, but having to split that activity among more communities, reveals an interesting dynamic. The number of interactions a user has with their $x^{th}$ ranked subreddit increases as we increase their number of subreddits. However, the percentage of interaction in their $x^{th}$ ranked subreddit does not change. With the exception of the 1st subreddit, the percentage of activity for a user in their $x^{th}$ ranked subreddit is broadly not affected by their overall number of subreddits. The third subreddit has approximately 10% of activity, the fourth approximately 6%, etc. This dynamic is remarkably consistent, although it is unclear if it generalizes outside of the COVID context, it would sync well with other such rules-of-thumb regarding social media activity such as exponential activity [9].

**Fig 6:**
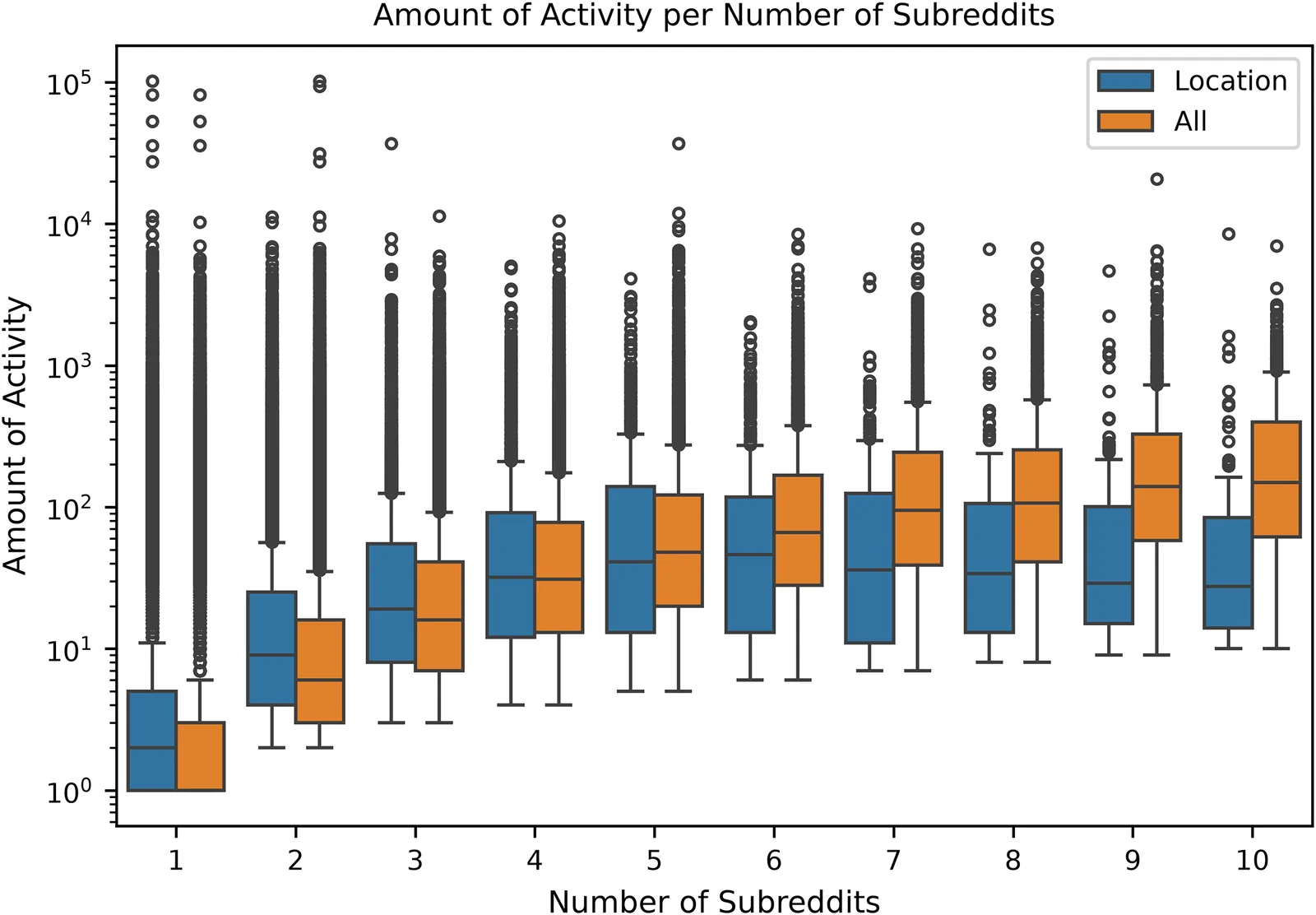
Amount of activity per number of subreddits. Distribution of number of total interactions for users in *x* subreddits for all data, and for only Location communities. Users with more active subreddits are generally more active across the board – they post and comment more than other users not just in more diverse places. This is a particularly dramatic shift from 1 to five active subreddits, and then levels off more. This perhaps suggests that in order to sustain participation in multiple subreddits, more activity overall is needed, as opposed to a hypothesis of equal amounts of activity split among more places. The direction of causality, however, is unclear. It is possible that more community interaction causes more activity. It is equally possible that more activity causes more community interaction. it is also possible that both are true. Note also that the shift is even more dramatic as users increase their Location subreddits from 1 to 5, but similarly levels off even more.

Additionally, for many users their most active class is very active, as we can see in Fig 5. While average activity does decrease as the number of communities increases, it slows to almost linear at about 40%. Similarly, the average amount of activity for the second largest class hovers between 20% and 30%, the third at about 10% and so on. This consistency is surprising. While we don’t have enough data to test this for all classes, if we restrict to just the Location class these numbers hold steady. We can also see that the median percentage of activity for the top ranked subreddit is very high for all numbers of subreddits, but while the percentage decreases as the number of subreddit increases, the total number of interactions actually goes up. That is, users with many subreddits are generally more active in their top subreddits. This increase in counts would suggest that users with more subreddits are generally much more active than users with fewer subreddits (Fig 6). In fact, this increase in activity outweighs the fact that the activity is spread across more subreddits, resulting in being even more active in each subreddit than users with more concentrated activity.

For a broader view of user behavior we define a null model of user overlaps. Suppose each time a user joins a community (by creating their first post or comment in a community), they join a random community weighted by its total number of users. That is, we suppose a world where users, instead of joining communities that interest them, join only communities based on their popularity. In this world, users are more likely to join more popular communities and less likely to join less popular communities with no other factors considered. With this assignment, we can then calculate the number of users we expect to be part of any two particular subreddits, based solely on popularity. Table 4 shows how many more or fewer overlapped users various classes have than we would expect given this model. Note that since this model allows for users to join the same community twice, we remove r/Coronavirus – the only community large enough for this to be a concern.

**Table 4:** Percentage of expected user overlap.

Most		Least	
%	Class	%	Class
3343	Skeptics/Skpetics	21	NSFW/Recovery
3277	Anti-Vax/Anti-Vax	44	NSFW/Disease Discussion
2455	Long Covid/Long Covid	48	Memes/Recovery
1950	General/Skeptics	51	Memes/Long Covid
1647	Anti-Vax/Location-Anti	52	Memes/Covid Activities
1587	General/General	60	Covid Activities/Disease Discussion
1566	General/Other	63	Covid Activities/Long Covid

A table of percentage of expected user overlaps (100% is as expected, higher is more than expected), for the 7 most under and over expected classes. First, we see that the most over-represented pairs are three of the same class pairs – where users are part of two subreddits of the same class. This, in conjunction with previous results showing that users are less concentrated in classes than expected means that these few classes, in particular, are distinct from other classes in their amount of self-overlap. For the pairs with fewer overlaps then expected, we primarily see NSFW and Meme accounts – this could be because users that participate in these use separate accounts for more “playful” activity or it could show a divide in that some users used Reddit as a more grounded discussion tool and some as a place for humor or fun.

There are a few interactions that stand out here. First of all, most classes are more likely to overlap with themselves than predicted – especially Anti-Vax, Skeptics, and Long Covid. That is, a user who is active in one subreddit in that class is far more likely to be active in another subreddit in that class than our prediction. One good example of this are Anti-Vax and Anti-Lockdown, which share ideological overlap and thus likely an overlap in community if not strictly topic. A less obvious case might be Disease Discussion and Skeptics – this shares topic overlap in a different way. Some overlaps are far less common than predicted. NSFW and Recovery are perhaps an unusual pairing that seems to be less common than expected, although in this case it is largely due to NSFW being a very insular class – few users are part of NSFW and another class. Activities During Covid and Memes is another unexpected unlikely pairing where it is less clear why this might be.

There are two additional things to note here. First, given the sheer number of users involved in this analysis, almost all differences are statistically significant even with corrections for the number of tests. Second, our size-based null model is based on total size of the community, including users who are part of only one community, whereas overlaps only use users with at least two active communities. This means a class might have strictly large or strictly small percentages, indicating a general tendency to be more or less insular as a class.

**Fig 7:**
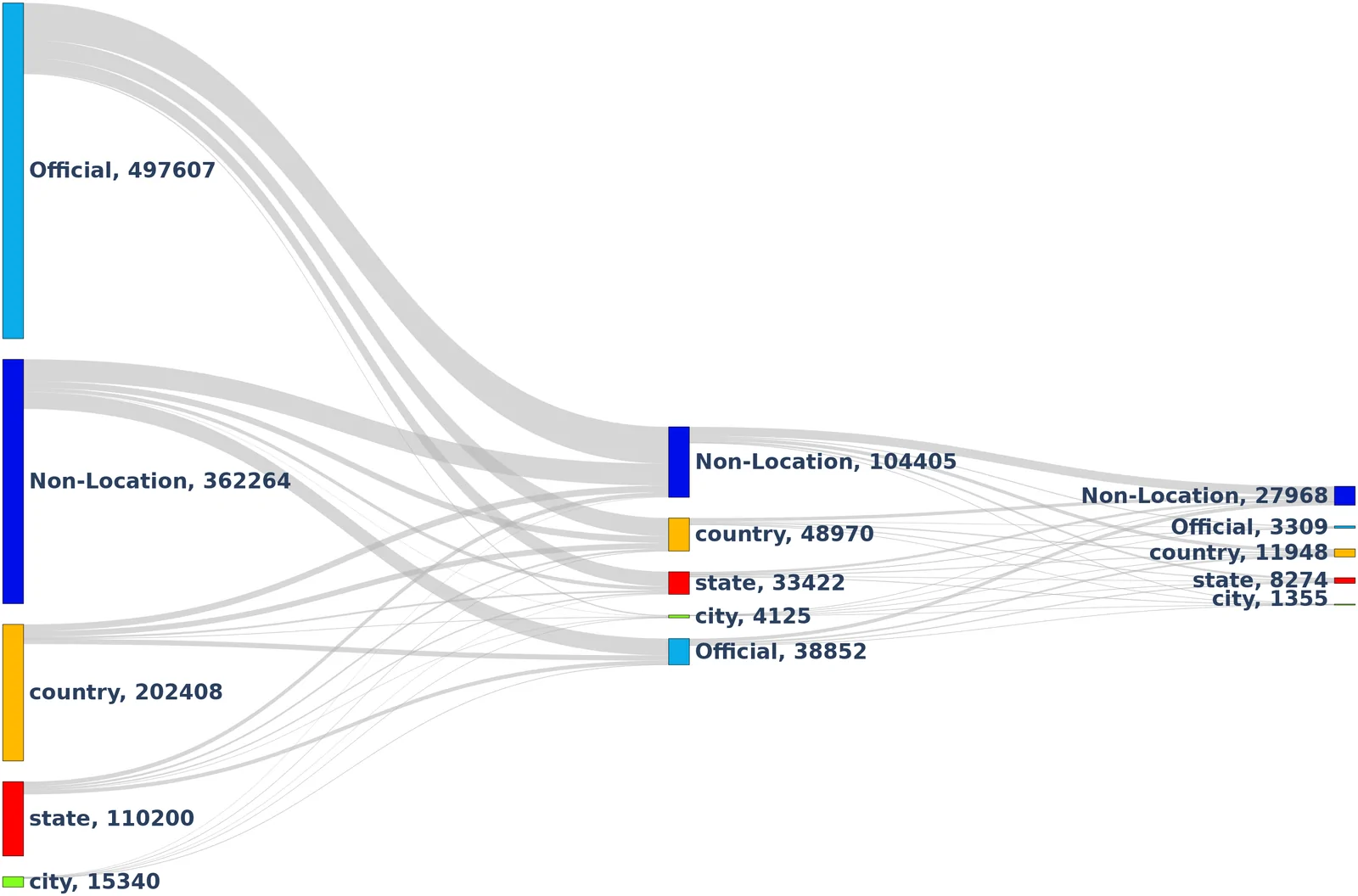
First three communities by class. Sankey diagram showing the class choices for the first 3 communities joined. Non-Location represents all subreddits that are not Location or Official. Note that most users join Official first, and then move to various Location communities, particularly country and state communities. This, again, suggests that Reddit’s attempts to designate an official community as a primary point of information sharing was highly successful – particularly with more shallow users with fewer additional subreddits. It also re-emphasizes that location played a very important role in users’ first few communities.

Given that many users participate in multiple classes, we then wonder if there is an ordering to the classes they join. Table 5 displays the count (and percentage of expected) for ordered overlaps, where an count in cell (*x*, *y*) represents a user who joined their first community in class *x* and then later joined their first community in class *y*. Expected, in this case, is gained by random ordering of joins rather than random assignment. Fig 7 shows the first three classes chosen by users in a Sankey diagram. As one might expect, Official is an outlier here – it is far more likely to join Official first. Similarly, more users join Recovery and then Long Covid than the reverse. However, this is also true for Skeptics, Long Covid and Skeptics, Recovery, perhaps suggesting a directional relationship between having Covid and being interested in skepticism. There is also a temporal aspect at play. Anti-Vax, a more recent class, is also less likely to be joined early across the board. Note also how much Location features in these early choices, although it is inline with expectations of overlap due to its sheer size.

**Table 5:** Percentage of expected user directional movement.

Most		Least	
%	Class	%	Class
204	Official/Anti-Vax	4	Anti-Lockdown/Official
201	Economy/Vaccine Discussion	7	Long Covid/Official
200	Location-Anti/Anti-Vax	7	Vaccine Discussion/Official
199	Official/Anti-Lockdown	8	Location + /Official
196	Official/Vaccine Discussion	11	Anti-Anti/Official
196	Official/Long Covid	16	Recovery/Official
194	Official/Anti-Anti	20	Location + /Location

A table of percentage of expected ordered overlaps for the 7 most over and under expected ordered class pairs. A count (*x*, *y*) represents a user who joined their first community in class *x* and then later joined their first community in class *y*. It is perhaps unsurprising, given our previous results, that most users begin with the official subreddit as their first point of contact and thus all other subreddits come later. In fact, nearly every pair includes the official subreddit. This emphasizes the role that this subreddit played not only for users who participated in only one subreddit, but additionally for repeat visitors. Additionally, the overwhelming priority on the official subreddit reinforces the role that Reddit played in shaping the discussion and suggests that similar efforts to designate and moderate a more ‘official’ forum could be highly effective in reaching users.

**Fig 8:**
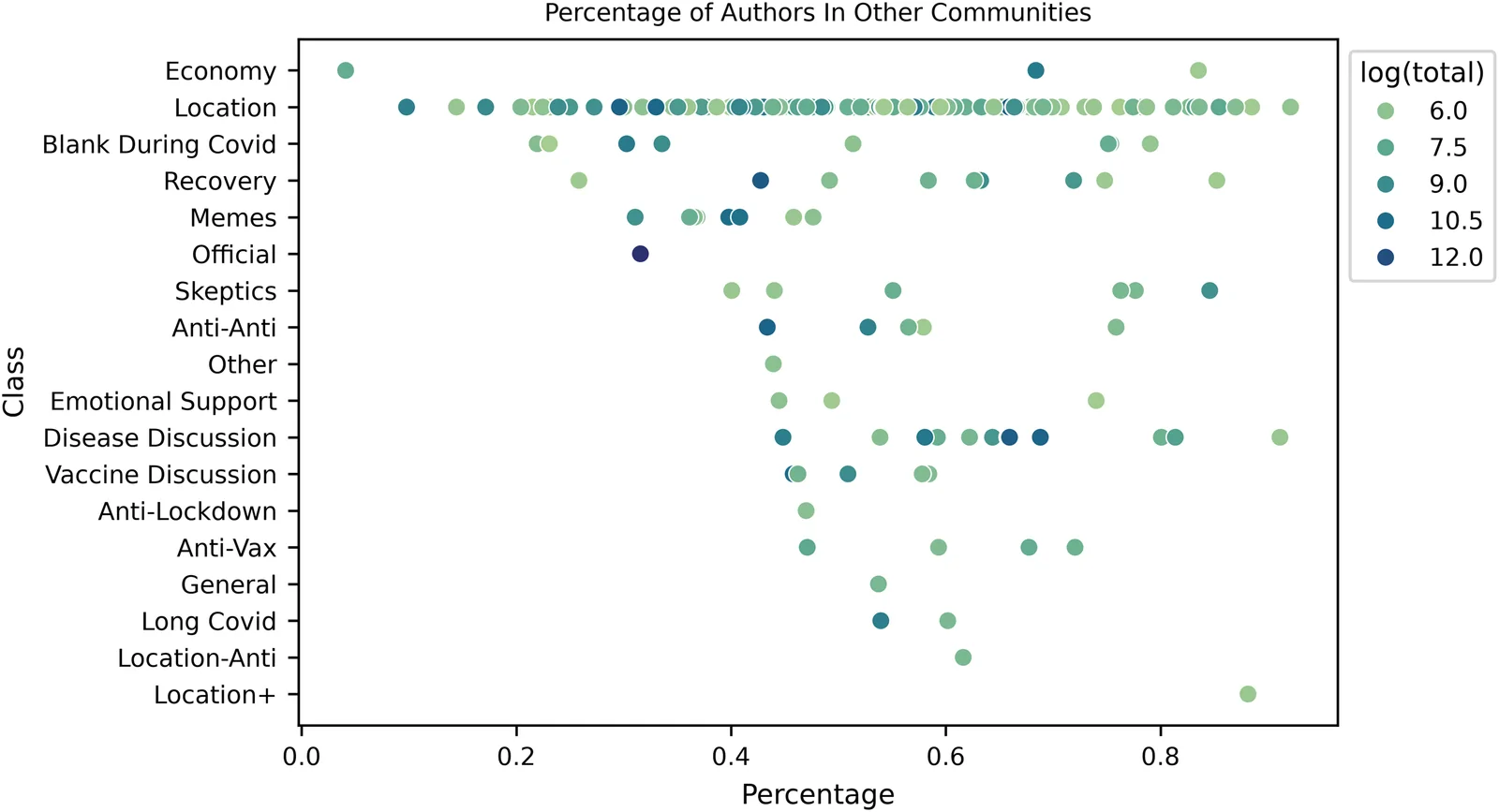
Insularity of subreddits. Insularity of various subreddits – higher percentage of user overlap means lower insularity – here the x-axis shows the proportion of authors in a subreddit that have participated in another subreddit within the class. We can also discuss cross-class insularity, which is the proportion of authors within a class that have participated in a subreddit in at least one other class. Color is size by author-count on a log scale. Note the wide disparity in insularity across communities and classes. Insularity is one way to measure community overlap, which has been shown to have strong impact on community survival and evolution [47]. Location, in particular, had very disparate insularity, with the larger communities generally having lower insularity – suggesting little user overlap between these larger Location subreddits (which were generally not geographically adjacent). However, insularity was otherwise generally quite low, with more than 40% of users in most classes belonging to at least one other subreddit in the class. This suggests that users would use Reddit for multiple purposes surrounding discussion on the pandemic – not solely one purpose. Contrastingly, the high insularity for Location, however, suggests the opposite. Users that were involved in Location subreddits did not participate outside of this class, suggesting they used Reddit solely for local discussion and not broader topics. Given the large percentage of users who were involved in Location subreddits, this could point to a divide between users who used Reddit for general discussion and those who used Reddit for local discussion.

These class tendencies brings up the question of insularity. How much does a class overlap with itself versus other classes? This value varies quite a lot by class (Fig 8). NSFW barely ever overlaps with other classes (almost all authors only exist in exactly one community). Skeptics, on the other hand, has remarkably low insularity as a class and for each community. This suggests that Skeptics is not merely an echo-chamber where users exist solely within its bounds but also interacts significantly outside of its own bubble. This is perhaps surprising, given previous work linking alternative communities to such echo-chambers [39]. Anti-Vax is more insular both in class and for the largest communities. This again underscores differences in the Skeptics and Anti-Vax communities. Anti-Lockdown appears somewhere in the middle, near many of our other classes. Location has remarkably variable insularity among the communities, but high insularity as a class. The variance in insularity among both classes and communities raises questions about how modularity-based community detection would behave in these circumstances – given that this method looks for density differences in connections, of which insularity is a good representative. It seems likely that many common community detection tools would struggle in this scenario, emphasizing the need for care when using such tools. While unsubstantiated in this experiment, it is possible that this pattern of high variance is common across similar set-ups of highly related but potentially conflicting communities, such as in Hessel et al. [45].

### Content

**Research question 3: How does posted content vary across communities?**

URLs are a unique opportunity to look at where users were drawing outside information from and how they are relating their community discussions to the larger internet. Analyzing this information flow, and in particular the information sources being used by various participants in the online space, is an essential part of understanding how social media impacted public activity and public opinion [28,48]. Particularly in a global emergency such as COVID, where information was sparse and spread rapidly, these URLs are an important part of understanding what shaped the flow of discussion and who users referenced and trusted.

**Fig 9:**
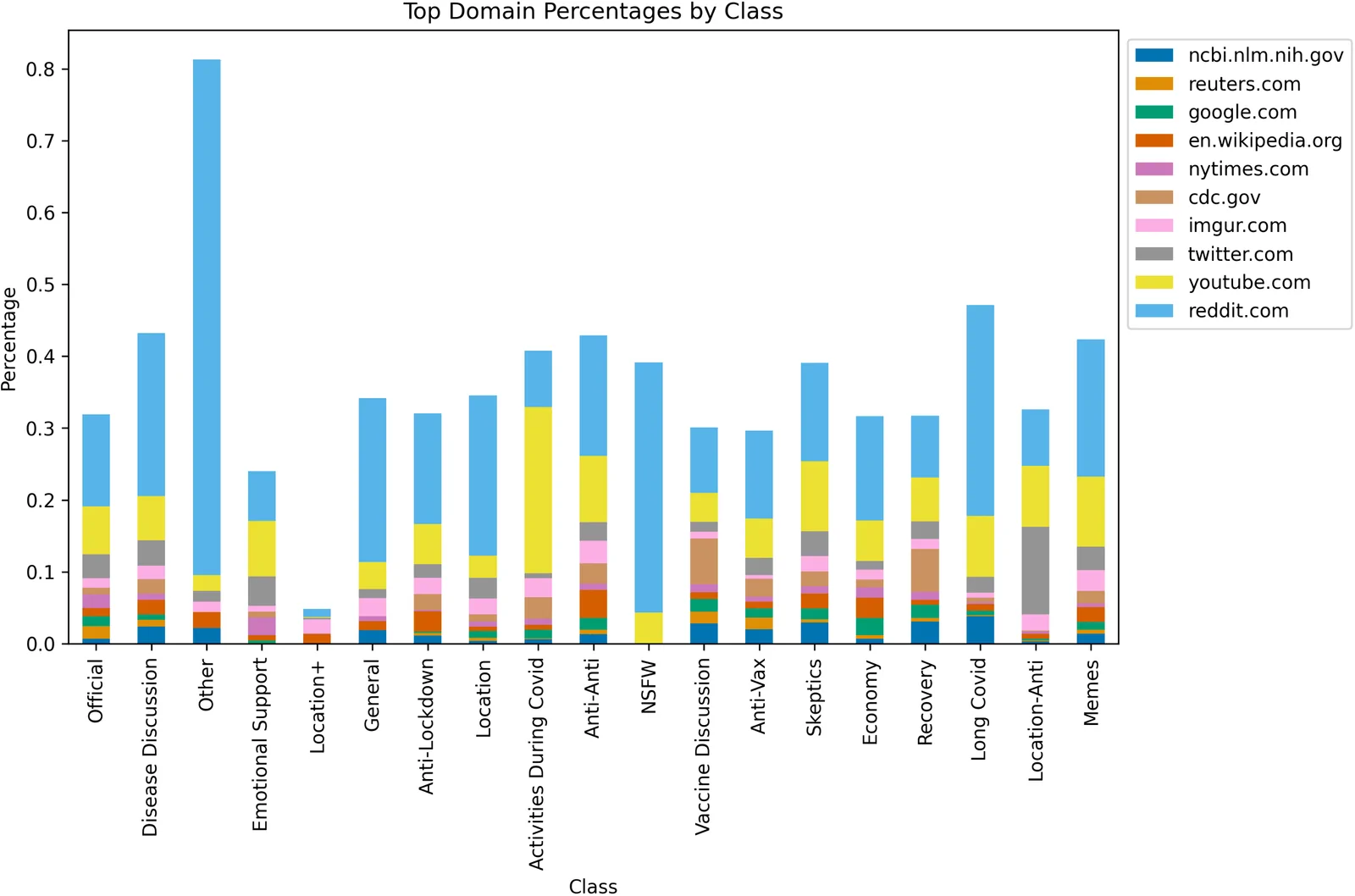
URLs in top domains. Percentage of URLs in various classes that go to one of the top 10 most common domains. Note that the most common link is, by far, Reddit itself. This is notable because it shows how efficiently information travels across Reddit, and how much Reddit discusses itself. This may encourage researchers to think of Reddit as highly interconnected, rather than as silos of communities. The other most common URLs include mostly major sources of information – social media and mainstream news sources which is perhaps as one would expect. Note also that various classes have preferences for various information sources. For instance, Location-Anti has a strong preference towards Twitter, Covid Activities prefers YouTube, and Wikipedia has an outsized influence on Anti-Lockdown, Anti-Anti, and Economy classes. This difference, despite the interconnectedness of Reddit, may harken to the different use cases of various information sources [13], but also is a strong indicator for where similar conversations are likely to happen and similar communities exist. Location+ is mostly dominated by github.com posted by a bot user that provides Twitter information.

If we take a closer look at the domains used, we can see that there are a few domains that dominate the URLs in our data set (Fig 9). Reddit.com, unsurprisingly, is the most common domain as users reference other content on the site. The next rest of the top 10 most common domains are a combination of social media sites, news organizations, and reference sites. However, different classes, again, use these domains differently. For instance, Activities During Covid references YouTube very heavily while Vaccine Discussion spends more time on CDC sites. However, the fact that these domains appear heavily in all our classes suggests that users are getting information from the same sources, regardless of the specific topic they are discussing.

**Fig 10:**
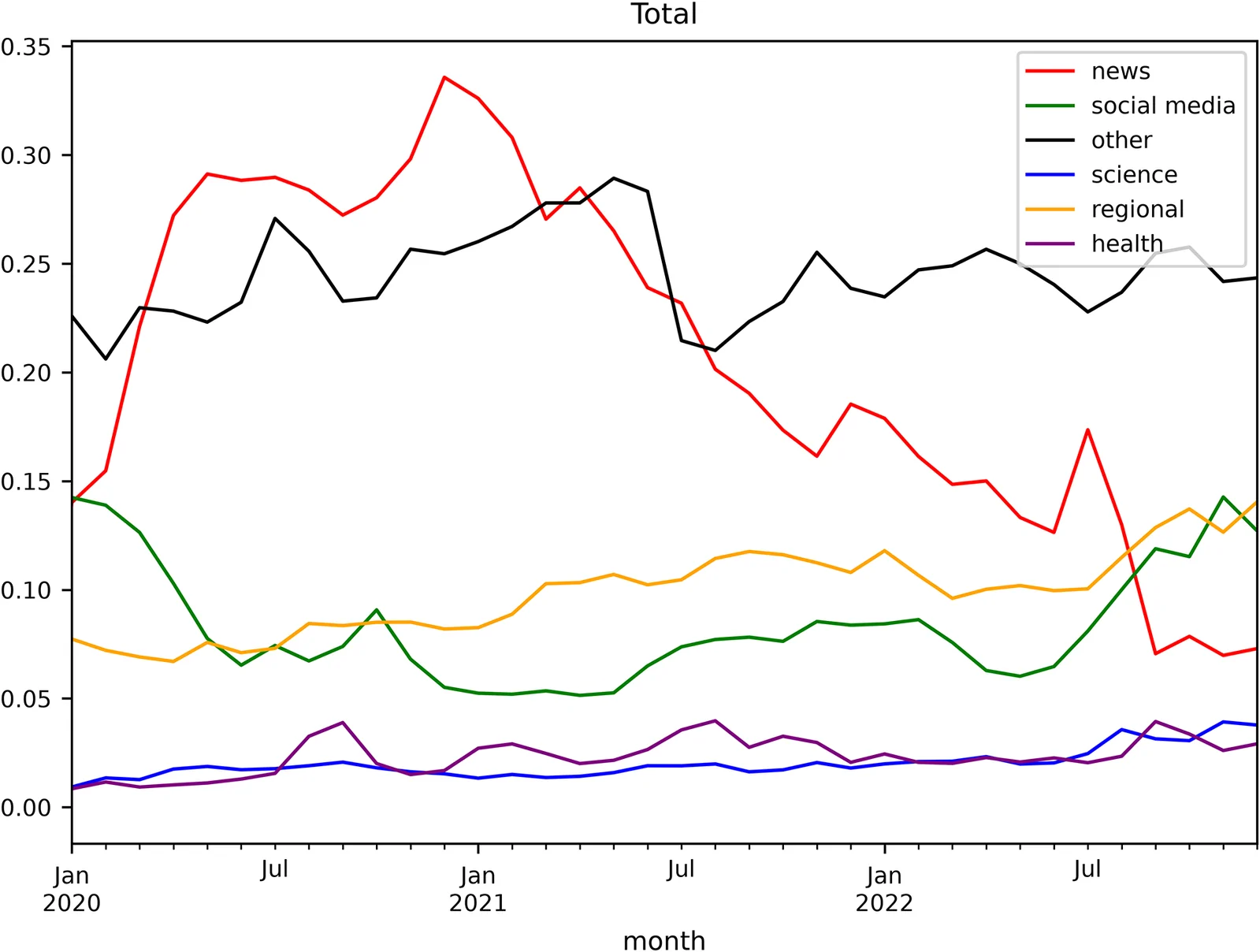
URLs by classification. The percentage of all URLs that lead to various types of websites. Note that news dominated early in the pandemic, but by 2021 began to fade in importance. Social media has an inverse trend, with more social media URLs at the beginning and end of our time frame. We also see a slight increase over time of regional URLs. This could point to an interesting difference from more short-term crisis studies, where social media is usually very important in the short-term and more traditional news sources become more important later [28]. Here, on a much larger time scale, we see the opposite effect.

Zooming out, we can also classify URLs by type [49] and analyze the popularity of these domains (Fig 10). Overall, news domains remained popular throughout the pandemic, fulfilling the subreddits’ role as community bulletin boards. However, popularity did shift over time. For instance, we see a spike in news URLs at the beginning of the pandemic before a long decline. This decline is largely due to Location communities phasing out news sources over time, while Official (the other major source of news URLs) maintains the popularity of news for the most part. However, regional (mostly referring to regional news) actually saw a slow increase in popularity, contrary to the more general news category. The ‘other‘ type’s most common domains are COVID trackers and sites like medarxiv that have medical science, which maintain popularity throughout. Note also that, with 2046710 distinct URL-author pairs, 67% are URLS posted once by an author and only 6% more than twice. This suggests both that spammers and bots are uncommon in our data set, but that repeat postings are not uncommon.

## Location

**Research question 4: How did the subset of geographically focused communities self-organize?**

Throughout this analysis, we have repeatedly found that the Location class is unique. It is by far the largest of our classes both in terms of activity and number of communities. It behaves differently in terms of users with multiple communities, and it has a fundamentally different structure. Not only is Location splintered into many communities by nature, but those communities have their own internal **vertical** organization that describes the type of geographic location they are attached to. We split that into five levels: ‘country’, ‘state’ (US sates), ‘city’ (US cities), ‘state_f’ (states outside the US) and ‘city_f’ (cities outside the US).

### Communities

Like most other classes, nearly all Location communities were created within a short period. These communities sometimes reference the same geographic areas – which we call **isomorphic** communities – but there is almost always one dominant community for a given location. Additionally, the creators of these subreddits tend to remain heavily involved in the community. While the conversation around COVID never died out during our time period, it did wax and wane. These fluctuations are often in line with three major waves of the epidemic: the initial wave, the Delta wave in mid 2021 and the Omicron wave in late 2021 and early 2022.

**Fig 11:**
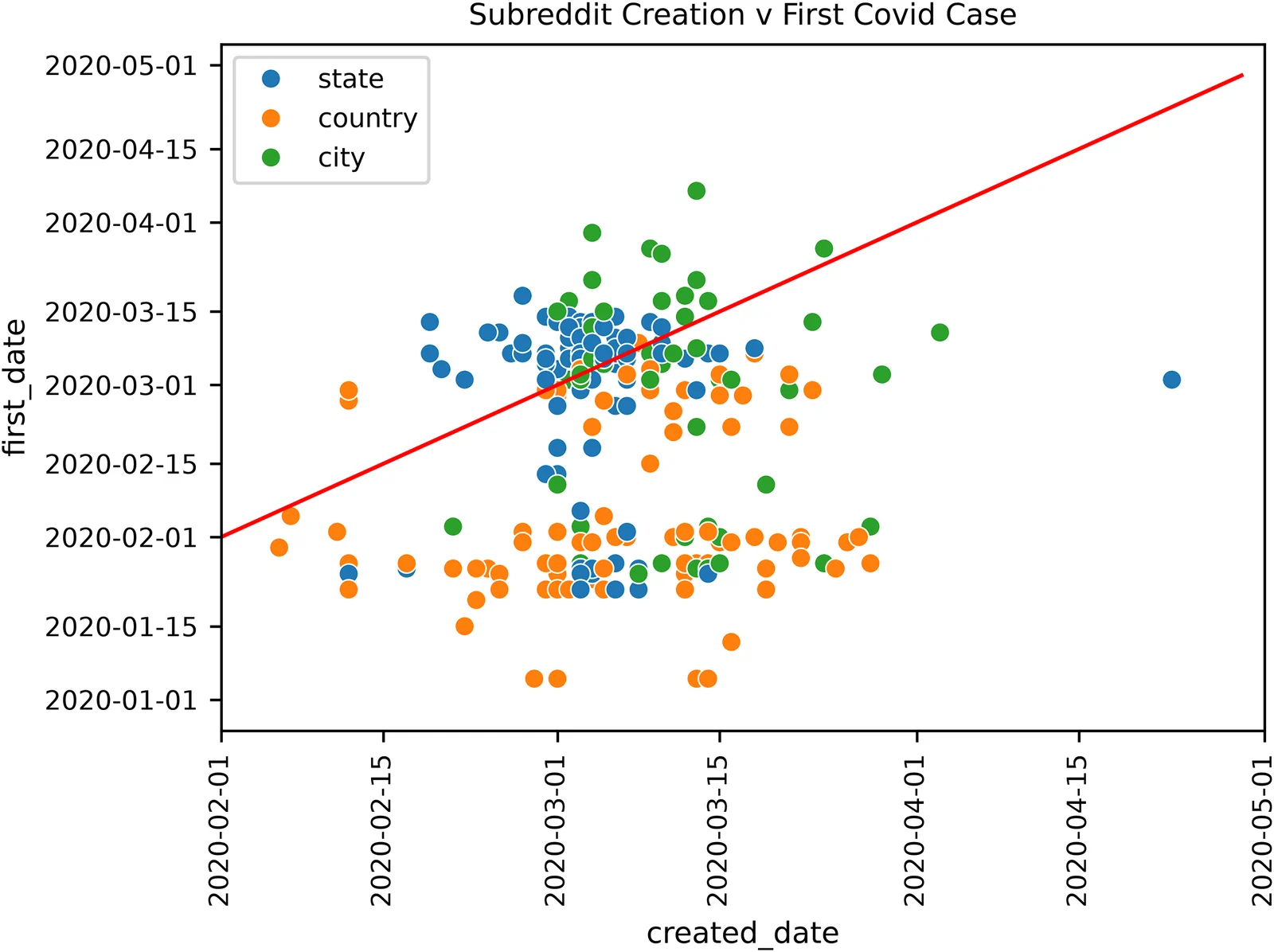
Date of creation of location subreddits. Date of creation of local subreddit vs date of first recorded COVID case. Subreddits above the line had a subreddit before the first case, those below had a COVID case before the subreddit was created. Note that most subreddits were created within one month – mid February 2020 to mid March 2020. Almost all state subreddits were created within a couple of day of the beginning of March. Most early subreddits were country subreddits, but it took longer for city subreddits to be created. However, creation date and the date of the first Covid case in the location are not highly correlated, suggesting that the impetus for creating a location-specific subreddit is not necessarily location-specific variables. This persistent difference in creation based on the size of the location is an effect not previously studied in the importance of place making and geographical social media [15].

Overall, the vast majority of Location subreddits were created right around when COVID became a global concern – when it began to pop up in countries world wide. It was declared a pandemic on March 11, 2020. Most subreddits were created between March 1st and March 15th, slightly predating the pandemic designation. As can be seen in Fig 11, the first communities were related to countries, with states and cities closely behind. This mirrored the dates of first COVID cases, with most cities and states creating communities before the first local case, but after the first case in the country.

The next obvious question is – who created these communities? There are 249 distinct users who created the 282 Location subreddits. Most users create only one subreddit. 19 users created more than one, and only 6 created more than two. The most prolific creator, cryptodude1, created 8 subreddits for 8 different states – all on March 1st and 4th. Only one of those subreddits became popular. The next most prolific, askcoronavirus, created 5 variations of subreddits for Michigan, mostly on February 24th, with only one that had any real activity. This pattern continues, with most creators who create more than one subreddit creating state subreddits. Additionally, creator involvement post-creation varies wildly for both Location and non-Location communities. While there is a natural decline in the amount of creator involvement as the size of the subreddit increases, among smaller subreddits (even into the thousands of posts/comments), some creators remain highly involved, accounting for almost all activity in the subreddit.

As mentioned previously, many locations had multiple subreddits. We call these “isomorphic” subreddits. These are most common for state subreddits, where only 15 states have one subreddit (and three have none). However, countries also frequently have isomorphs. The US has 10 isomorphic subreddits, and Canada and India each have nine, the UK and ‘Europe’ each have 7, and Australia has 6 (Fig 13). These are the locations with the most isomorphs. They also, notably, include a large portion of the English-speaking world.

**Fig 12:**
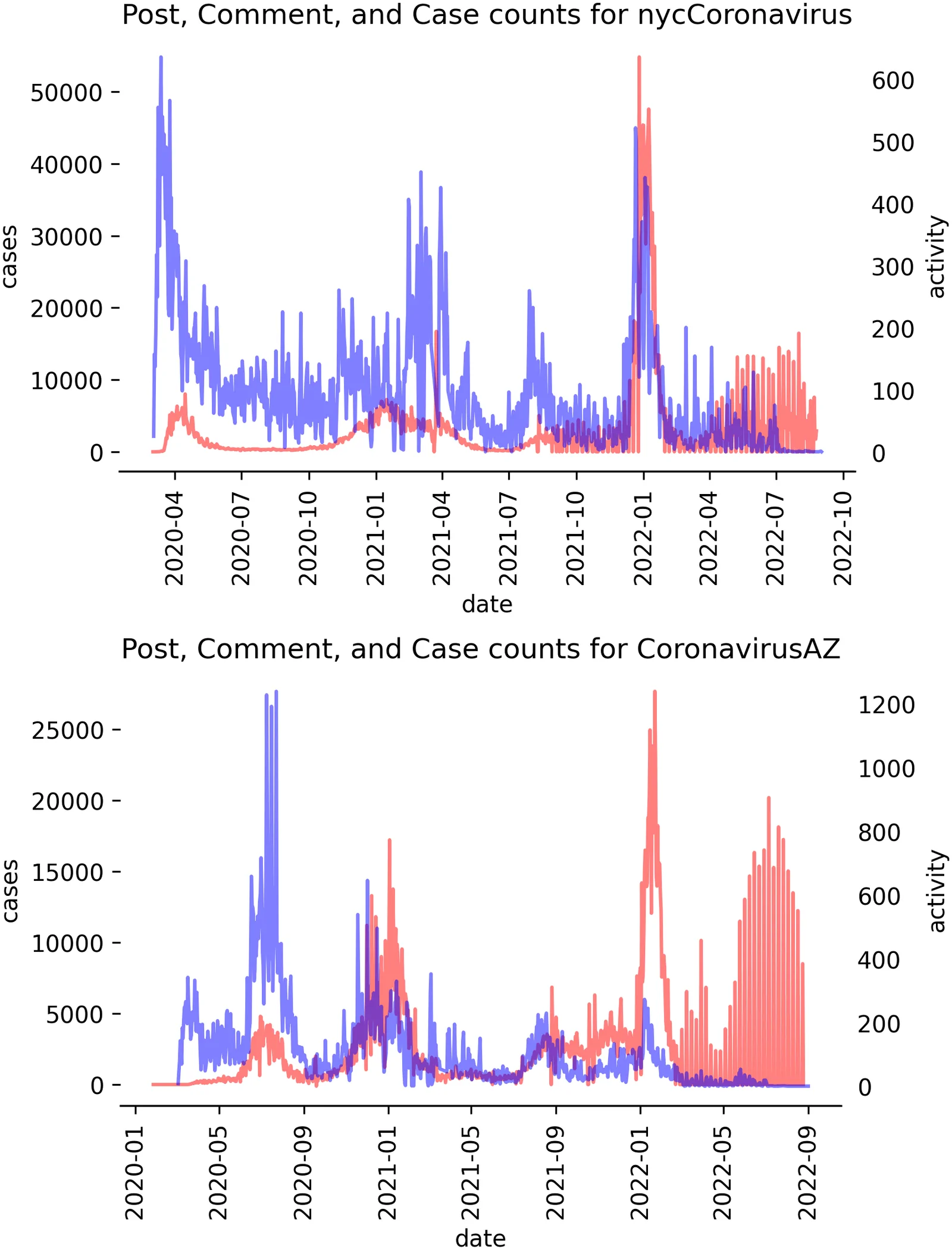
COVID cases versus amount of activity. Red is number of cases per day, blue is number of posts and comments per day. NYC and Arizona had two starkly different reactions to COVID cases but maintain some similar themes. In both cases, online activity began with a burst even before Covid began to its first local spike. This activity quickly quieted. We also see an outsized reaction to early spikes of Covid – with strong increases in online activity. However, by the end of 2021 this pattern begins to fade. Subsequent spikes, despite being larger than earlier ones, are met by less online activity. This is far more pronounced for Arizona, however, than NYC, as online activity nearly ceases by early 2022. This demonstrates strong online fatigue in both cases, but much stronger in Arizona. It also demonstrates strong reactions to both early local spikes, and the onset of the pandemic. However, Arizona had much less reaction to subsequent spikes than NYC and online activity ceased much sooner despite a higher relative case count. The strong contrast of location-specific information impacting the activity in, but not creation of, location-based subreddits is an interesting dynamic. It could suggest a shift in understanding of the pandemic as most communities were created prior to large local impact, but perhaps in anticipation of need. However, the activity itself reflects on-demand use rather than anticipatory use.

**Fig 13:**
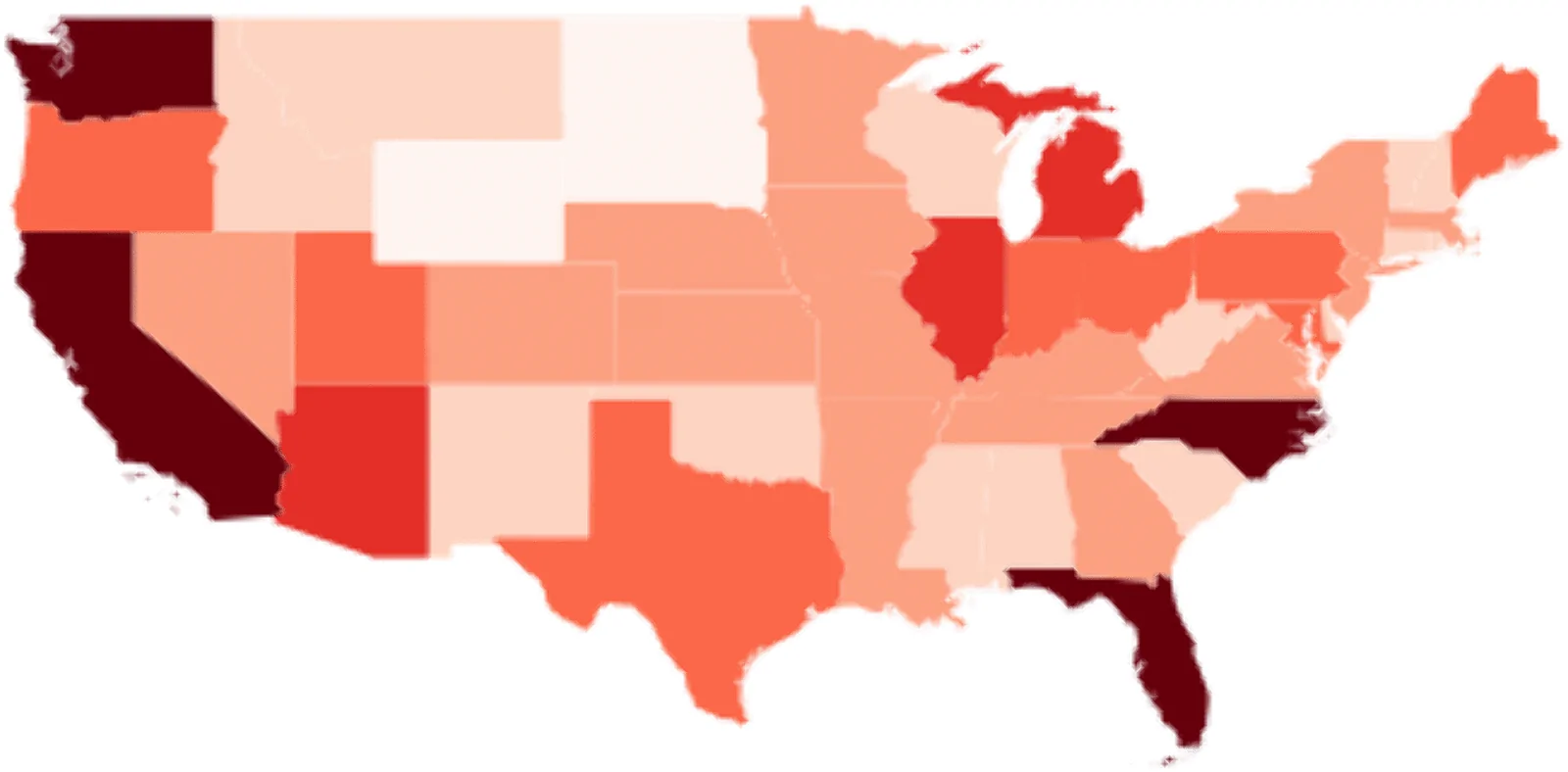
Isomorphic state subreddits. Most states in the US had at least two subreddits and up to six, darker states have more isomorphs [50]. Three states had none (the Dakotas and Colorado). In most cases, the first isomorphic subreddit to be created became the dominant one and the other others quickly died off, suggesting little need for additional state specific communities and a more cohesive community structure. Redundancy in online communities is an area of active study, and the fact that redundancy is so rare in location-based subreddits is perhaps unusual [51].

Additionally, in most cases the first isomorphic subreddit is created within two weeks of the original subreddit, and often much sooner. Most isomorphic subreddits are created by different users in close succession, mirroring the burst of subreddit creation seen in other classes. Using our previous definition of dominance, where the most popular subreddit is at least twice as active as the next most popular subreddit, of 142 distinct locations, only 6 lack a dominant subreddit. For the 56 locations where there are at least two competing subreddits, in 35 instances the dominant subreddit is the first created, suggesting a bias towards initial creation not seen in other classes.

In terms of activity, while activity varies from community to community, we do see close correlation with the spread of Covid Fig 12. This change in activity reflects the creation dates, in that both are closely tied to the changing status of the pandemic in the locality discussed in the community. However, this pattern falls off quickly. This cements the importance of these communities early in the pandemic, whereas their importance seems to fade somewhat over time as they are less reactive and less active than before. This initial reaction to look for location-based communities may be an interesting avenue to explore for research into information diffusion in times of uncertainty or emergency, as it may provide avenues for improved reach. It also emphasizes that Location as a category behaves differently from our other categories.

### Content

Location subreddits are less topic-focused, so in addition to URL analysis we also introduce keyword analysis. These two items provide key insight into the topics being discussed and the sources of information relied upon for those discussions. These items reflect the theme of political differences, particularly with respect to what URLs are shared in different states. It also reflects differences in topics and outside information sources both vertically and horizontally across geographic locations. However, there are also some common themes. URLs again take the role of information diffusers, as we see most popular domains relate to information directly related to the disease. Given this, the political differences are even more stark, as they could suggest differing sources of trusted information as well as differing interests.

News sources are, particularly during the pandemic, inherently political. So it is worth asking if that political bent is evident in the URLs used in these online discussions. We test this by checking the correlation between the popularity of a URL in a given state and the percentage of the state that voted for Biden in the 2020 presidential election. This can then be compared to a null model in which we randomize the states in which URLs appeared – in this null, we would randomly shuffle URLs such that URLs are assigned randomly to states, but the overall number of URLs for each state remains the same. This would give us a baseline for comparison to a world where any factors about states themselves is ignored. Checking the 200 most popular domains among state subreddits, we find 15 domains with strong correlation with Democratic votes and only one domain (thetennessean.com) with strong correlation with Republican votes (Table 6). This suggests that Democratic states tend to use more popular domains more frequently than Republican states. We confirm this by checking to see how many domains it takes to account for random samples of the URLs used in different states. Democratic states generally have significantly fewer domains than Republican states (average ~350 versus an average of ~400). Even when accounting for number of URLs, Democratic states use the same domains significantly more frequently than Republican states, suggesting more variation of the domains in use in Republican-leaning states. This could reflect a “mainstream” versus “alternative” influence. This difference again emphasizes the different experiences users had with Covid online if they lived in different locales. Despite being on the same social media site, by organizing into local communities, users see starkly different sources of information. Note also that not all URLs are posted in good faith. When we observe URLs commonly posted by users that are later banned or suspended, we see that many are political (Table 7). Given these accounts were later banned, this suggests the users were not participating in good faith or were otherwise behaving counter to community norms. As such, more political URLs are perhaps also used provocatively rather than to engage in discussion. We see a bias towards conservative-leaning domains in this case, which may reflect the generally liberal bias in most of Reddit.

**Table 6:** Domain correlation with 2020 democratic votes.

Domain	Corr.
nature.com	0.71
medrxiv.org	0.58
webmd.com	0.54
thelancet.com	0.53
bmj.com	0.50
thelancet.com	0.48
wbur.org	0.45
bostonglobe.com	0.41
bbc.com	0.53
nejm.org	0.52
amazon.com	0.50
theatlantic.com	0.46
instagram.com	0.45
pubmed.ncbi.nlm.nih.gov	0.45
ncbi.nlm.nih.gov	0.40

Correlation of domain prevalence in a state subreddit with the percentage of Democratic votes in that state for the 2020 election, for domains with p-values less than 0.01. These domains tend towards mainstream news sites and scientific medical information sites – sites that were popular throughout the pandemic. Note that only one domain showed the opposite correlation.

**Table 7:** Domains from banned/suspended users.

	% active	% banned	% suspended	domain	URL count	authors count	class
0	14.0	6.9	79.2	cutt.ly	159	31	other
0	24.2	6.0	69.7	linktr.ee	99	35	computers
0	39.5	54.4	6.1	huffpost.com	114	32	news
0	43.2	51.0	5.8	in.reuters.com	206	34	news
0	45.4	30.8	23.8	www.thegatewaypundit.com	130	40	news
0	46.4	38.4	15.3	www.breitbart.com	209	55	news
0	47.3	44.8	7.9	www.aier.org	165	41	other
0	47.4	45.5	7.1	theweek.com	310	81	news
0	48.1	47.4	4.5	sports.yahoo.com	133	32	news
0	48.8	50.1	0.3	www.cebm.net	340	78	science

User statuses for domains with at least 30 uses and 10 authors, where fewer than half of authors are active. Percentages are percent of instances of URL where user has a given status. Most of these domains are either spam or political, and several such as breitbart, thegatewaypundit, and aier and conservative political sites. This suggests that users use these domains to make drive-by political points rather than actively participate in the community.

Another method of analyzing content at scale is tracking keywords. We identify keywords by using the Fighting Words [52] method, which identifies words that differentiate two sets of strings – that is the words that most differentiate speech between two groups of text based on log-odds with a dirichlet prior. By comparing state subreddits, we can analyze major differences in regional conversations. Logically, most states have a lot of location-specific terms including cities or politicians. Additional terms such as ‘vaccinated’, and ‘cvs’ may suggest some topics differences, but overall most states seem to have similar discussion topics.

While we see that terms vary geographically, there is also a strong temporal aspect. One interesting case the use of the terms ‘coronavirus’ and ‘covid’. The use of ‘coronavirus’ spikes at the beginning, but ‘covid’ slowly increases in popularity and eventually overtakes ‘coronavirus’, reflecting a change in the dominant terminology. ‘Lockdown’ versus ‘quarantine’ tells a different story. Both terms are popular during similar time periods, but ‘lockdown’ shows up much more in non-US subreddits than ‘quarantine’ does. Terminology changes can represent both changes in how speakers represent something or changes in who they are being influenced by. The strong temporal aspect of these terms suggests rapid shift in some aspect of this – and that this shift is captured in online forums relatively cleanly. The terminology used during the pandemic is a point of interest for many in public health, as understanding how this terminology changes, how to influence it, and what advantages and disadvantages different naming conventions bring all are important to responding to public health concerns [53].

### Users

We see that users that interact with more communities are much more active overall, posting and commenting far more than those with fewer communities. However, users also seem to have a ‘dominant’ subreddit where they spend most of their time – and users have about 40% of their interactions occur within this dominant subreddit regardless of how many subreddits they interact with. These additional communities also show a tendency to broaden their communities vertically before horizontally.

The vast majority users are part of only one subreddit, and only a handful of users are part of more than ten subreddits (Table 3). For those who are active in only one subreddit, 1717520 are part of a country subreddit, 458629 part of a state subreddit, 41650 part of a city subreddit compared to 2045922 in Official. Location subreddits become less popular as they become more specific. Interestingly, if we look at how much of a subreddit’s user-base also participates in other subreddits, we see a very large percentage across the board. The number of subreddits with very large overlap percentages decreases as we look at larger and larger subreddits, but not nearly as much as we would expect if we scrambled user membership via a null model with bipartite randomization -(in this bipartite null, we randomly re-assign users to new communities such that we hold steady the number of users in each community and the number of communities for each user but scrambling actual membership. This both shows that users with just one subreddit are more likely to participate in large subreddits (possibly because they are easier to find, or because smalls subreddits to not satisfy the need of a user alone), and that there is significantly more user overlap than would be predicted at random in general.

**Fig 14:**
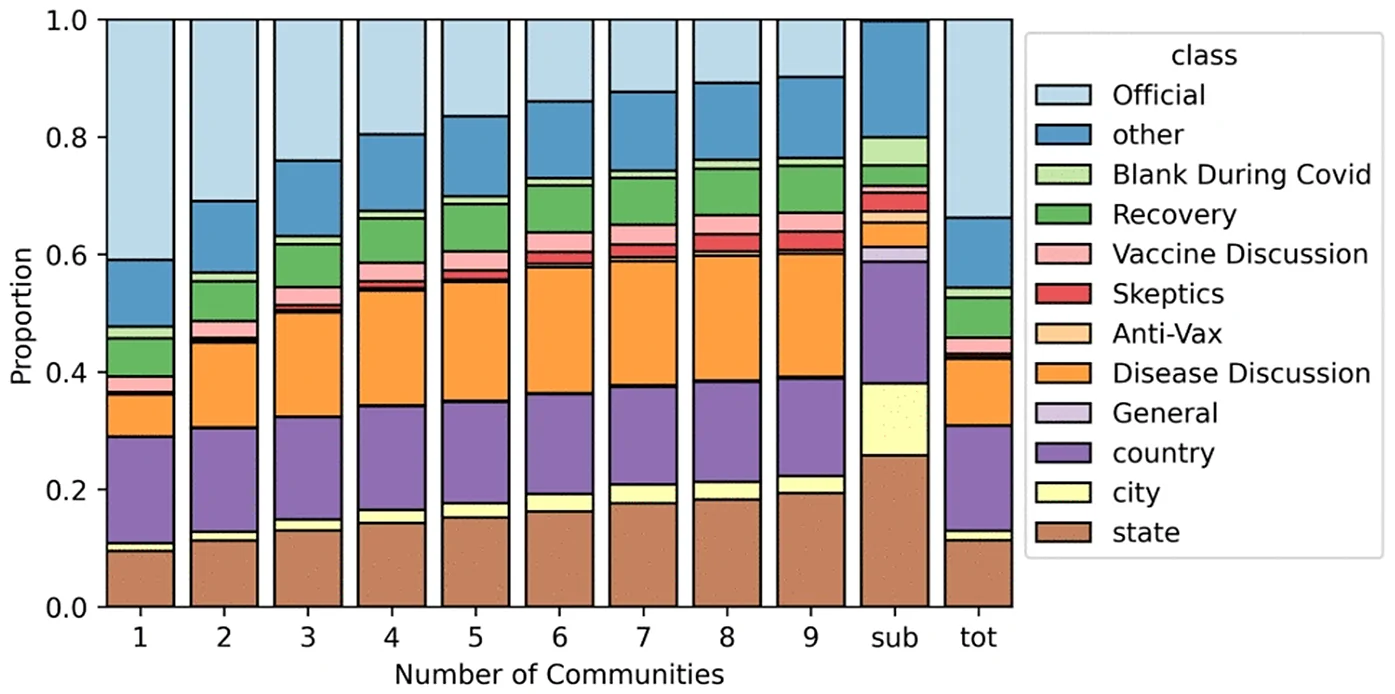
Percentage of subreddit participation by class. The percentage of subreddits participated in for each class, across all users that have participated in *x* subreddits. The total bar is an overall percentage for all users, and the sub bar is the percentage of subreddits at each level. Note that there is only one Official subreddit, but it has a very outsized influence. Note also that the number of General subreddits and state subreddits increases dramatically with the total number of communities, suggesting that users with few communities focus on the Official community – although not exclusively so, but those who join additional communities tend to be more even in their distribution.

If we compare this to users that participate in multiple subreddits, we can see that this is a higher percentage of General subreddits, but also a significant increase in the number of state and city subreddits (Fig 14). However, it seems that, apart from those with only one subreddit, the overall percentage of country subreddits is fairly stable. The large increase in the percentage of state subreddits suggests that most users who participate in multiple subreddits are looking for additional communities at the state level – we now must ask why. The rapid decrease in Official is likely due to there being only one official community.

**Fig 15:**
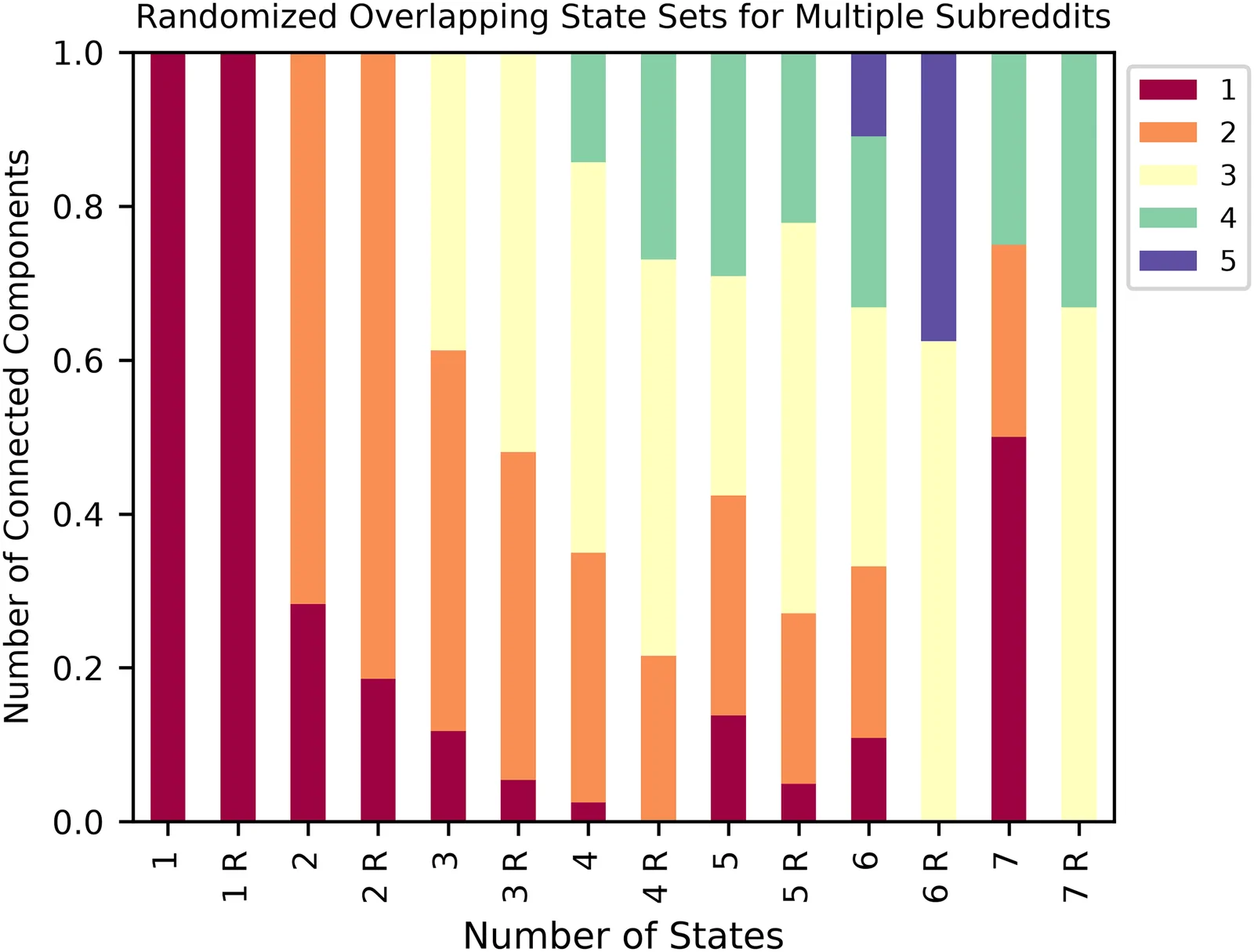
Distinct state geographic groupings. For users with *x* non-isomorphic state subreddits, how many distinct groups of geographically adjacent states these states form. The bars with “R” have had the state-user pairs randomized as a null model – this null is also a bipartite randomization as described earlier. This shows that, when a user is part of multiple state subreddits, those states tend to be in contiguous blocks of adjacent states.

If we look at where the states are geographically located (Fig 15), we can see that in most cases users are very likely to choose states that are geographically close, more so than if we randomized user-subreddit pairs. This could express a desire for regional subreddits that are not constrained by specific state boundaries.

Moving beyond just the horizontal specialization of states, this level notation allows us to ask questions about the implicit vertical hierarchy. If a user is part of a city subreddit, are they also part of the relevant state and country subreddit? A user who is in a city subreddit is in the corresponding state or country subreddit about 25% of the time. The number is similar for state subreddits.

**Fig 16:**
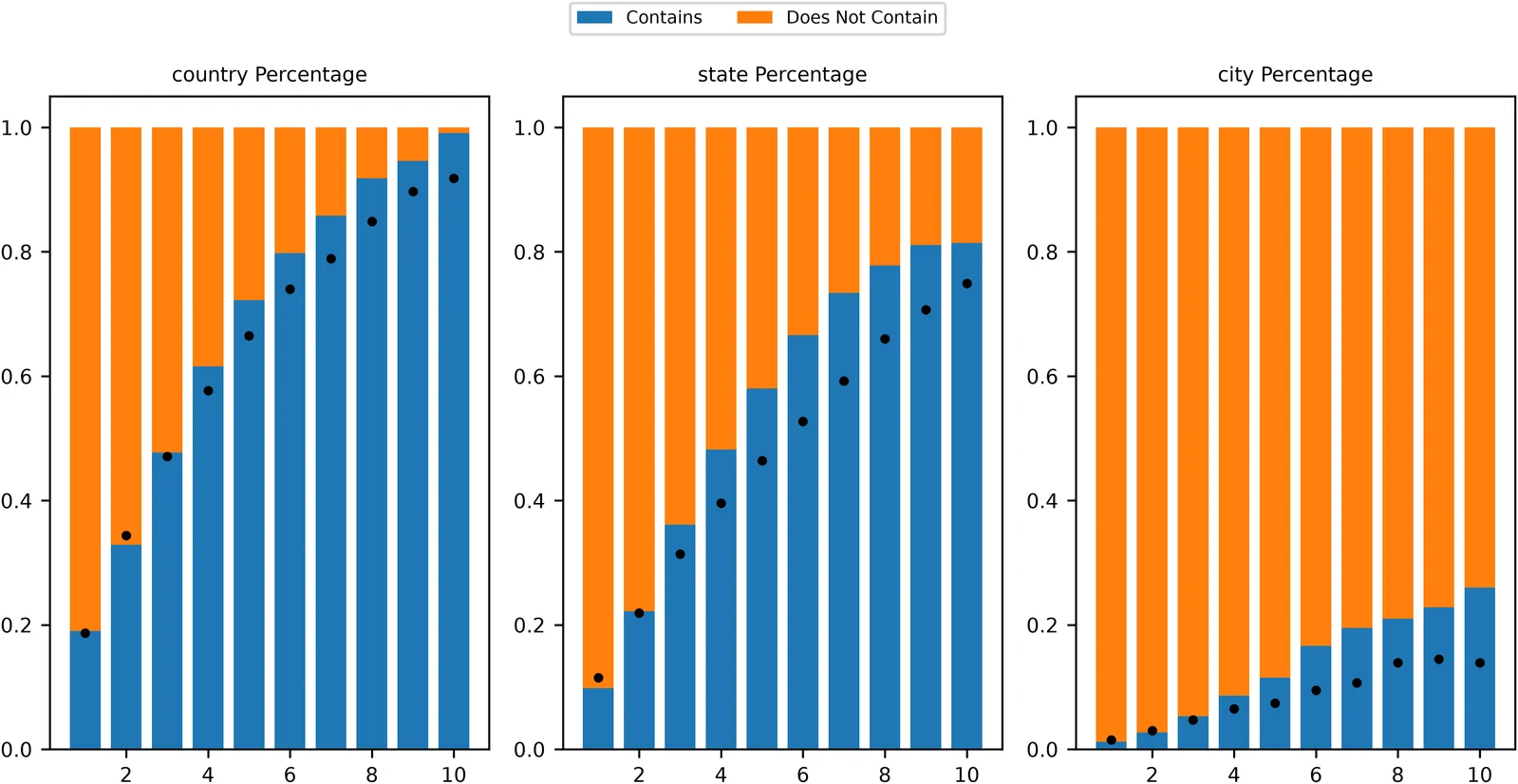
User participation in vertical location levels. Number of users with *x* subreddits where their subreddit list contains or does not contain at least one subreddit of a given level. The black dots represent a null model of bipartite user-subreddit pair randomization. Overall, more users participate in these subreddits than expected at random, given our pair randomization that means that at random we would expect location participation to be more concentrated, with more users part of multiple subreddits of the same level. The likelihoood of a user participating in a country or state subreddit tracks closely to the random percentage, however users become more likely than expected to be part of a city subreddit as the number of subreddits they participate in increases.

We then again turn to look at the differences between users that are part of few or many subreddits (Fig 16). In this case, we can see that the percentage of users who have a subreddit of a particular level is somewhat dependent on the number of subreddits they are part of total. Both country and state percentages seem to increase in a roughly exponential way, but city percentage appears more linear. Also, while country and state percentages are somewhat close to our random baseline, city subreddits deviate from those baselines, suggesting the bottom level may be fundamentally different.

If we take subreddits to be nodes and connect those nodes if they share some minimum percentage of users, then we have a small simple graph of subreddit overlaps. This graph has one giant component, with a highly connected core and a few periphery nodes. However, if we increase the minimum percentage, we get a more focused view of the core of this graph. Another concrete way to testing the geographic influence on community membership, is to ask if the number of geographically significant connections remains the same for randomized graphs. We use both the configuration model and the k-core model [54] as null models and check how many connections there are between adjacent and non-adjacent states.

For adjacent states, the true graph has about 1.9% of edges as edges between adjacent states. For the configuration model, the average drops to 1.0%, but for the k-core model we average 1.6% - close to the original. This suggests that much of the adjacent state connections are reflected in the core structure of the graph – small clusters of interconnected states. However, for non-adjacent states the average percentage is much higher for both null models (13% and 15%, respectively) than the original (3%), which reflects a huge geographic influence in state connections – far fewer connections between non-adjacent states than we would expect at random. Rather, these edges may instead be part of more vertical connections than expected at random. Finally, for isomorphic states, fewer than 1% of edges are edges between isomorphic subreddits for both random models, whereas nearly 8% of edges are edges between isomorphic subreddits in the real graph. These measurements reinforce the fact that geography is highly influential in the communities users are a part of.

**Fig 17:**
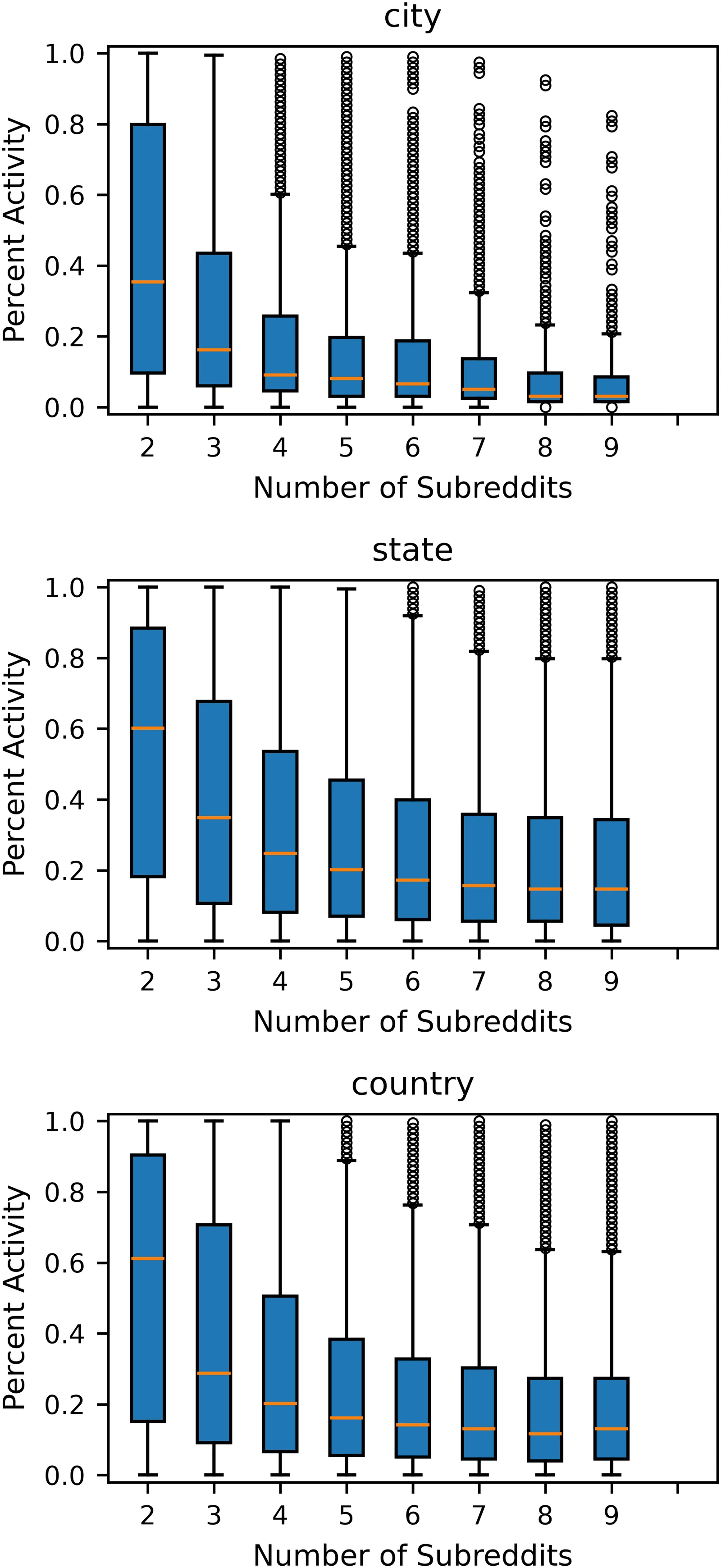
Activity in various location levels. Percentage of activity in a given level for users with at least one subreddit in that level and *x* subreddits in total. Note the wide variation in how much time users spend in their location subreddits. Note also that state and country levels tend to see very high levels of activity, and that the median amount of activity is actually above 0.5 for two subreddits, and never falls much below 0.2 regardless of additional subreddits. This suggests that even very active users spend significant amounts of their time in state/country subreddits.

Looking at activity through the lens of vertical levels of geographic organization — across different spatial scales — one interesting point is that some levels have more activity than others (Fig 17); country and state have generally high levels of activity, for instance. Also, while one might expect the percentage of activity to decrease as the number of subreddits increases (since more subreddits of other levels may be introduced), this is not strictly the case. The median percentage of activity does not decrease much past the 3rd community, while the 75th percentile does decrease somewhat more dramatically. This is likely closely linked to the amount of activity overall for users with additional subreddits which increases a generally, but tapers off much faster for Location subreddits (Fig 6).

## Conclusion

Our analysis of the spectrum of different COVID communities on Reddit allows us to study how a large volume of online activity rapidly self-organized around an abruptly emerging topic of global scale. Against this backdrop, we analyze the content discussed in these communities in the form of URLs and keywords, the behavior of users in terms of which communities they participate in and how much they participate, and the self-organization of the online discussion into these communities. We have structured this analysis to include the existing themes that guided the organization of these communities, including political, skeptical, and geographical ones, with geography playing the quantitatively largest role in organizing the communities.

COVID-related communities on Reddit formed rapidly and settled into distinct topical classes—intermediate groupings of subreddits that function like tectonic units, each with its own structure and boundaries. A significant portion of these communities were geographically focused, accounting for nearly 40% of activity and engaging over a third of users. Most communities emerged within weeks of the pandemic’s declaration, with little reorganization afterward, except in response to later developments like vaccines and long COVID. User participation patterns reveal that individuals tend to concentrate their activity within a few favored subreddits, with consistent proportions of engagement across their top choices. However, insularity varies widely, even within the same topical class, challenging assumptions about uniform modularity and complicating community detection methodologies.

Content posted within these communities also reflects distinct patterns. URLs serve as a powerful lens for understanding external influence and authority, with anti-vaccine communities disproportionately referencing official sources like the CDC. Over time, geographically linked communities showed a marked decline in references to national and international news, favoring more localized sources. Within these location-based groups, political affiliation influenced domain sharing: Democratic-majority states leaned toward scientific sources, while Republican-majority states favored more region-specific outlets. The rapid formation of these communities led to isomorphic subreddit structures, where one dominant subreddit typically emerged per region. Users also tended to cluster in geographically and hierarchically adjacent communities, such as city and state-level subreddits, reinforcing spatial proximity in digital engagement.

While the COVID pandemic provided a unique opportunity to study these geographically specialized communities at a large scale, it is by no means the only place where this geographic specialization exists. Many of the themes we discuss in this work are applicable to other, non-COVID sets of communities and it would be interesting to understand the influence of the COVID topic. Similarly, this analysis is primarily US focused, and it would be interesting to produce analysis at similarly fine-grained resolution for the hierarchy of geographies in more non-US-based communities. Additionally, while this work focuses only on active participation of users, expanding the analysis to readership would provide additional context.

## Supporting information

S1 File Covid_subs_classified (1). (CSV)
